# Improving gut health and growth in early life: a protocol for an individually randomised, two-arm, open-label, controlled trial of a synbiotic in infants in Kaffrine District, Senegal

**DOI:** 10.1136/bmjpo-2022-001629

**Published:** 2024-02-27

**Authors:** Benjamin Momo Kadia, Marietou Khouma, Doudou Sow, Babacar Faye, Anouschka S Ramsteijn, Beatriz Calvo-Urbano, Modou L Jobarteh, Elaine Ferguson, Paul Haggarty, Joanne P Webster, Alan W Walker, Claire Heffernan, Stephen J Allen

**Affiliations:** 1 Department of Clinical Sciences, Liverpool School of Tropical Medicine, Liverpool, UK; 2 Service de Parasitologie-Mycologie, Faculté de Médecine, Université Cheikh Anta Diop, Dakar, Senegal; 3 Service de Parasitologie-Mycologie, UFR Sciences de la Santé, Université Gaston Berger, Saint Louis, Senegal; 4 Rowett Institute, University of Aberdeen, Aberdeen, UK; 5 Department of Pathobiology and Population Sciences, Royal Veterinary College, University of London, London, UK; 6 Department of Population Health, London School of Hygiene and Tropical Medicine, London, UK; 7 London International Development Centre, London, UK

**Keywords:** growth, gastroenterology, microbiology, parasitology, molecular biology

## Abstract

**Introduction:**

Infants exposed to enteropathogens through poor sanitation and hygiene can develop a subclinical disorder of the gut called environmental enteric dysfunction (EED), characterised by abnormal intestinal histology and permeability. EED can contribute to stunting through reduced digestion and absorption of nutrients, increased susceptibility to infections, increased systemic inflammation and inhibition of growth hormones. EED can be apparent by age 12 weeks, highlighting the need for early intervention. Modulating the early life gut microbiota using synbiotics may improve resistance against colonisation of the gut by enteropathogens, reduce EED and improve linear growth.

**Methods and analysis:**

An individually randomised, two-arm, open-label, controlled trial will be conducted in Kaffrine District, Senegal. Infants will be recruited at birth and randomised to either receive a synbiotic containing two *Bifidobacterium* strains and one *Lactobacillus* strain, or no intervention, during the first 6 months of life. The impact of the intervention will be evaluated primarily by comparing length-for-age z-score at 12 months of age in infants in the intervention and control arms of the trial. Secondary outcome variables include biomarkers of intestinal inflammation, intestinal integrity and permeability, gut microbiota profiles, presence of enteropathogens, systemic inflammation, growth hormones, epigenetic status and episodes of illness during follow-up to age 24 months.

**Discussion:**

This trial will contribute to the evidence base on the use of a synbiotic to improve linear growth by preventing or ameliorating EED in a low-resource setting.

**Trial registration number:**

PACTR202102689928613.

**WHAT IS ALREADY KNOWN ON THIS TOPIC?**

- Environmental enteric dysfunction (EED) reduces the digestion and absorption of nutrients, and increases susceptibility to infections and systemic inflammation, contributing to growth faltering in children.
- EED can appear as early as 12 weeks of age, and measures including exclusive breast feeding, nutritional supplements and improved water, sanitation and hygiene have not been sufficient to prevent EED and subsequent growth faltering in at-risk populations.
- Supplementation with a synbiotic may enhance colonisation resistance against enteropathogens and improve gut health.

**WHAT THIS STUDY HOPES TO ADD?**

- Evidence of whether administration of a synbiotic in early life improves linear growth in infants in a community exposed to poor hygiene and sanitation.
- Evidence of the effect of a synbiotic on biomarkers of gut health, enteropathogen colonisation, systemic inflammation, epigenetic status and growth.

**HOW THIS STUDY MIGHT AFFECT RESEARCH, PRACTICE OR POLICY**

- May build on the evidence base for causal relationships between gut health and growth.

## Introduction

Globally, stunting affects about 149.2 million children under 5 years of age, with 40% of affected children residing in Africa.^1^ Stunting is a complex process that manifests physically as significantly impaired linear growth, defined by a length-for-age z-score (LAZ)/height-for-age z-score more than 2 SDs below the WHO Child Growth Standards median.^2 3^

Recent large cluster randomised trials in Bangladesh,^4^ Kenya^5^ and Zimbabwe^6^ reported a limited impact of water, sanitation and hygiene (WASH) interventions and provision of food supplements on linear growth. Several studies have, however, demonstrated that growth faltering is associated with an enteropathy termed environmental enteric dysfunction (EED).^7 8^ EED is a subclinical disorder that results from damage to the intestinal mucosa by pathogenic microbes.^7 8^ It is characterised by villous atrophy, intestinal inflammation, and a ‘leaky’ intestinal mucosa.^9^ The consequences are reduced nutrient digestion and absorption, and systemic inflammation that reduces the production of growth hormones.^10 11^ EED can occur as early as age 12 weeks, and despite exclusive breast feeding.^10^ Biomarkers in stool and blood have been proposed as non-invasive and accessible indices of EED.^9^

One potential target for novel interventions to prevent or reduce stunting is the diverse and numerous collections of microbes (‘microbiota’) that colonise the gut of infants. The gut microbiota is critical for the development of the gut and other organs, mucosal and systemic immunity, and protection against gastrointestinal infections through a process termed ‘colonisation resistance’.^12–14^ However, the development of the gut microbiota in early life can be perturbed by factors such as caesarean section delivery and exposure to pathogens and antibiotics.^15^

Synbiotics, which are a combination of (1) prebiotic(s) and (2) probiotic(s), are one type of intervention with microbiota-modulating potential. Prebiotics are substrates that selectively promote the growth and activity of beneficial host micro-organisms, thereby conferring a health benefit.^16^ Probiotics are live micro-organisms which, when administered in adequate amounts, can confer health benefits to the host.^17^ In a large trial in India, a synbiotic given for just 7 days in newborns, nearly all of whom were exclusively breast fed, significantly reduced sepsis, pneumonia and skin infections.^18^ This landmark study showed that synbiotic administration is acceptable to mothers, feasible at scale and may have important health benefits. However, the study did not report effects on gut health or linear growth.

The primary objective of this trial is to determine whether supplementation with a synbiotic during the first 6 months of life improves linear growth of children at age 12 months. The trial will also assess the effects of the synbiotic on biomarkers of intestinal inflammation and integrity, systemic inflammation, gut microbiota maturation, levels of growth hormones, gut colonisation with enteropathogens, host epigenetic status relevant to gut and general health and growth, episodes of illness and linear growth up to age 24 months.

## Methods and analysis

### Trial design

Senegal Synbiotic (SENGSYN) is an individually randomised, two-arm, open-label, controlled trial. The protocol for this trial has been developed in accordance with the Standard Protocol Items: Recommendations for Interventional Trials guideline. The trial is part of the research conducted within the UK Research and Innovation Global Challenges Research Fund (UKRI GCRF) Action Against Stunting Hub (AASH) (https://actionagainststunting.org/), an interdisciplinary research consortium investigating the precursors of child stunting to inform programmes and policies to reduce the global burden of this condition. Infants born to women recruited during pregnancy in Kaffrine, Senegal in the AASH observational study will be assessed for eligibility and recruited to participate in the SENGSYN trial.

Recruited infants will be randomised to receive either a daily supplement of a synbiotic in the first 10 days of enrolment and then weekly up to 6 months of age, or no intervention, with the children followed up to 24 months of age. Detailed anthropometry will be conducted at birth and months 3, 6, 12, 18 and 24; stool samples will be collected at 1, 6 and 24 months; and blood samples will be collected at 6 and 24 months. Assessments of morbidity and breast feeding are conducted weekly up to 24 months of age (table 1).

**Table 1:** Schedule of enrolment, intervention and assessments

	Study period										
	Enrolment/allocation	Post allocation									Close-out
Time point	Birth	1 month	2 months	3 months	4 months	5 months	6 months	9 months	12 months	18 months	24 months
Enrolment											
Eligibility screen	X										
Informed consent	X										
Random allocation	X										
Intervention											
Intervention arm		Synbiotic daily for 10 days then weekly until 6 months									
Control arm		Routine care									
Assessments											
Weekly morbidity diary		X	X	X	X	X	X	X	X	X	X
Body weight, breast feeding	X	X	X	X	X	X	X	X	X	X	X
Other anthropometry		X		X			X	X	X	X	X
Stool pathogens/microbiota/ biomarkers		X					X				X
Blood biomarkers							X				X
Epigenetic status in saliva		X									X

The trial follows the Standard Protocol Items: Recommendations for Interventional Trials protocol.

### Study setting

The trial is based in Kaffrine District, Senegal. The total population is 257 696 inhabitants. The population of children under 23 month is 17 780, and the prevalence of low birth weight (BW) is 17%. Data from 2015 indicated a prevalence of stunted children under five of 26.8%.^19^ The district has 28 health posts or clinics and 32 health huts administered by the Kaffrine Health Centre. The AASH will recruit participants in seven clinics, and women participating in the AASH study will be targeted for the recruitment of their newborns in the SENGSYN study. More details on the study setting will be provided in the supplement paper on the overview of study design, data collection and management procedures of the AASH.

### Participants

Pregnant women recruited into the AASH observational study will be given an information sheet about this synbiotic trial at recruitment (online supplemental file 1). The women will be further approached by study staff at health facilities or in their homes within 3 days of delivery to determine whether their newborn meets the inclusion criteria and if she/he does, to obtain informed consent for participating in the SENGSYN trial (see online supplemental file 2)

10.1136/bmjpo-2022-001629.supp1 Supplementary data

10.1136/bmjpo-2022-001629.supp2 Supplementary data

Inclusion criteria are

- Singleton newborn.
- BW or current weight (if BW not known) of ≥2000 g.
- Healthy infant who is breast fed and has taken at least one breastfeed well.
- Age 1–3 days.

Exclusion criteria are

- Multiple birth (eg, twins, triplets, etc).
- Presence of any acute illness in the newborn (eg, fever and receiving treatment with antibiotics).
- Congenital abnormality that might be life-threatening or might impair growth.
- Infant with potential contraindication to synbiotic (eg, suspected immune suppression and cardiac abnormality).
- Mother unlikely to stay in study area for the duration of the trial.
- Any health- or study staff concerns regarding safety to participate in the trial.

Infants of women known to be HIV positive and without known immunosuppression are eligible to participate in the trial.

### Randomisation and allocation

The trial statistician will prepare a computer-generated random allocation sequence using R-coding, with blocks of random size stratified by clinic, allocating newborns to one of two study arms:

- Arm 1: Labinic synbiotic every day for 10 days and then weekly to age 6 months.
- Arm 2: control group, usual care with no supplement.

Participants (infants) will be allocated at a ratio of 1:2 in the intervention and control arms, respectively. The allocation sequence will be held by an independent pharmacist in Senegal who will prepare sequentially numbered, sealed, opaque envelopes according to the random allocation sequence. The random allocation sequence will be concealed from all other members of the research team including staff allocating newborns to the trial arms and laboratory staff analysing the biological samples. Each envelope will be opened sequentially following recruitment of each newborn and will contain a card indicating the trial arm. The trial arm will then be linked to the infant’s unique AASH identification number. Mothers/carers who decline for their infant to participate in the SENGSYN study will continue in the AASH observational cohort.

Following participant allocation, study staff will assist the mother/carer in administering the first dose of the synbiotic to infants in the intervention arm.

### Intervention

The trial will explore the impact of the Labinic synbiotic, composed of a prebiotic (BENEO Orafti Synergy1; 50% oligofructose/50% fructooligosaccharide, 200 mg) plus three live bacterial strains (*Lactobacillus acidophilus* NCFM, *Bifidobacterium infantis* Bi-26 and *B. bifidum* Bb-06; total of 5 billion organisms/dose (Biofloratech, Walton-on-Thames, Surrey, UK). The synbiotic supplement is a powder in capsules with one dose per capsule. The contents of the capsule can be sprinkled directly into the infant’s open mouth before feeding. Study staff will visit daily for 9 days after the initial dose and then weekly to age 6 months (32 doses in total) to supervise the administration of the synbiotic and record adherence. Administration will be repeated once if the infant vomits within 30 min of synbiotic administration. In infants taking antibiotics, including HIV-exposed infants taking daily co-trimoxazole, the synbiotic will be administered where possible at least 4 hours before/after the antibiotic or between doses to minimise the effect of the antibiotic on the synbiotic microbes. Antibiotic usage will be recorded during dosing visits and morbidity follow-up. Infants randomised to the control arm will receive the same follow-up as infants in the AASH observation cohort. All infants will receive routine care as per national guidelines and will have contact details and mobile phone numbers of study staff for assistance in case of infant illness.

### Outcomes

All infants will be followed up to 24 months of age with anthropometry, biological sample collections (blood, stool and saliva), and morbidity assessment conducted at scheduled time points (table 1).

#### Primary outcome

The primary outcome of the trial is linear growth at age 12 months assessed by LAZ. Regularly calibrated equipment for anthropometry will include a Model 876 SECA/infant scale, UNICEF stadiometer/infantometer, Lufkin WP601 measuring tape and Holtain skinfold calliper. Measurements will be conducted in duplicate with a third measurement taken if the first two measurements do not agree within a specified amount. Weight will be recorded to the nearest 100 g and height, length, mid-upper arm circumference, head circumference, triceps and subscapular skinfolds, and knee-to-heel length to the nearest 0.1 cm. Measurements will be used to determine z-scores using the WHO growth standards for LAZ, weight-for-length (WLZ), triceps-for-age and subscapular-for-age. Children will be classified as stunted or wasted if their LAZ or WLZ is <−2 SDs, respectively.

#### Secondary outcomes

The secondary outcomes are impact of synbiotic consumption on the presence of enteropathogens and parasites in stools, biomarkers of gut health in stool and blood, biomarkers of systemic inflammation, growth hormones, faecal microbiota composition and epigenetics markers. Stool samples collected at 1, 6 and 24 months will be analysed using multiple methods including bacterial culture, quantitative PCR and ELISA for the following:

- Enteropathogens: *Salmonella*, *Shigella* and pathogenic *Escherichia coli*.
- Enteroparasites: helminths (*Ascaris lumbricoides*, *Ancylostoma duodenale*, *Necator americanus*, *Trichuris trichiura* and *Strongyloides stercoralis*) and protozoans (*Giardia lamblia*, *Cryptosporidia parvum*/*hominis* and *Entamoeba histolytica*/*dispar*).
- Intestinal inflammation: myeloperoxidase.
- Intestinal permeability: α_1_-antitrypsin.
- Maturation of gut microbiota, assessed using Illumina-based 16S rRNA gene amplicon sequencing.

Blood samples collected at 6 and 24 months will be analysed for

- Chronic inflammation: alpha-1-acid glycoprotein.
- Acute inflammation: C reactive protein.
- Gut mucosal integrity: intestinal fatty acid binding protein.
- Growth hormones: insulin-like growth factor (IGF)-1 and its carrier protein IGF-binding protein 3.

Saliva samples collected within 1 month of birth and at 18–24 months will be analysed for epigenetic status including markers of gut and general health and growth.

Procedures for the transportation, storage and handling of the aforementioned biological samples will be detailed in other protocols of the supplement (for blood and stool samples, see protocol titled ‘Assessment of the role of gut health in childhood stunting in a multi-site, longitudinal study in India, Indonesia, and Senegal: a UKRI GCRF Action Against Stunting Hub protocol paper’, and for saliva samples, see Epigenetics protocols and methods).

Infants who develop adverse events that may be due to the study intervention will be identified by parents/carers contacting study staff or at follow-up visits. If appropriate, participants will be referred to the hospital for evaluation and treatment according to local guidelines. In the case of a serious adverse event (SAE), subjects will be referred to an appropriate health facility for management. All SAEs will be reported to the in-country principal investigator or an assigned representative within 24 hours of the research staff becoming aware of it. The information reported will include the nature of the event, date of onset, severity, corrective therapies given, outcome and causality (ie, unrelated, unlikely, possible, probably and definitely). The responsible study clinician will assign the causality of the event. SAEs that are unexpected and are at least ‘possibly related’ to the study intervention will require expedited reporting within 24 hours of the in-country principal investigator or assigned representative becoming aware of it. This will be a maximum of 48 hours after the event occurred or the study team was made aware of the event (including the 24 hours required for the field staff to report to the principal investigator/representative). Additional information will be sent within 14 additional days (full SAE report) if the reaction had not resolved at the time of email notification.

### Sample size determination

The sample size calculation is based on observing a clinically meaningful improvement in linear growth in the intervention arm. In the WASH Benefits study in Kenya, the control group (n=1431) at median age of 12 months (range 2–18 months) had a mean LAZ of −1.13 (SD=1.13).^5^ To observe a 25% improvement in LAZ at age 12 months (ie, LAZ=−0.8475, SD=1.13) with 80% power, α=0.05 and with 1:2 group allocation would require 189 infants in the intervention group and 378 in the control group. We plan to allow for 20% loss to follow-up by recruiting 236 to the intervention group and 472 to the control arm. Based on previous studies, we consider that this number of infants will also be sufficient to observe meaningful differences in biomarkers between the study arms.^8 10 12 20^

### Data management and statistical analysis

#### Data and sample management and governance

Data and sample management will follow procedures established by the AASH. This includes a data sharing strategy that will build on the guiding principles for scientific management and stewardship and Concordat on Open Research Data to ensure equitable access to data. Data will be transmitted via secure file transfer protocols between Senegal and hub partners in the UK, Indonesia and India. A data monitoring and evaluation committee (DMEC), a trial steering committee (TSC), research ethics committees of Liverpool School of Tropical Medicine (LSTM) and London School of Hygiene and Tropical Medicine, and Senegal National Ethics Committee for Scientific Research will oversee the conduct of the trial. The trial sponsor, LSTM, will review the trial at initiation, during the study and at trial close-out with additional monitoring visits as required. The trial may be stopped or suspended by the sponsor at any stage due to any arising safety concerns or following the recommendation of the DMEC and/or TSC.

### Statistical analysis

Analysis of the primary and secondary endpoints will be based on intention to treat. Mean/median values of biomarkers, the proportion of infants with abnormal biomarker concentrations, gut colonisation with enteropathogens and the proportion of infants/children with episodes of illnesses will be compared in the two arms at 1, 6 and 24 months. Additional analysis of the primary and secondary endpoints will also be presented according to adherence to synbiotic administration. Data will be analysed using generalised linear models with treatment as the only predictor, generating the estimates of treatment effects and their 95% CIs. For outcomes with repeated measurements, the linear model will have a binomial distribution and logit link function for binary outcomes and a normal distribution and identify link function for continuous outcomes. Mean differences between the two treatment arms together with 95% CIs will be derived from the appropriate linear model. Missing data will be treated as missing completely, and no imputation of primary or secondary endpoints will be made. Adjustment for covariates and inclusion of interaction terms will be performed as appropriate. More details on statistical analysis methods using secondary variables will be described in other papers in the supplement.

### Patient and public involvement

There was no involvement of patients or the public in the design of this trial.

### Protocol version

The protocol version is V.2.0, 12 June 2020.

## Discussion and conclusion

Childhood stunting affects many children in vulnerable populations, particularly in Africa, while interventions targeting WASH and nutrition have had limited success improving linear growth. Evidence has accumulated for suboptimal gut health and EED contributing to early life growth faltering. This clinical trial aims to evaluate the effectiveness of a synbiotic in improving linear growth in early life, as a potential affordable and scalable public health intervention.

There are many potential prebiotic, probiotic and synbiotic preparations on the market, with different mixtures of substrates and micro-organisms. However, the limited knowledge of their effects on the gut complicates the selection of specific products for evaluation in clinical trials, including for the prevention or amelioration of EED. Identifying probiotics with specific antipathogen properties relevant to EED is difficult given the limitations of in vitro and animal models in mimicking the complex environment of the infant’s gut and the numerous enteropathogens that may contribute to EED.^10^ Colonisation resistance is mediated through multiple mechanisms, and some are likely shared among *Lactobacillus* and *Bifidobacterium* species, including production of fermentation acids and acidifying the gut lumen.^15^ Probiotics may have additional beneficial effects in EED through reducing intestinal inflammation and permeability.^21^ Prebiotics may supplement human milk oligosaccharides, the natural prebiotics in breast milk, with additional carbon sources to promote the growth of putatively beneficial microbes such as bifidobacteria.^22^ Prebiotics may also serve as decoy receptors for enteropathogens.^23^

Lactobacilli and bifidobacteria are supplied routinely to about 70% of preterm infants nursed in neonatal units in Germany,^24^ based on their potential to prevent necrotising enterocolitis^18^ and late-onset sepsis.^5^ A recent survey reported that Labinic was used routinely in preterm infants in seven neonatal units in the UK.^25^ The Labinic probiotic has been associated with a significant decrease in necrotising enterocolitis and late-onset sepsis in preterm infants with no episodes of sepsis due to *Lactobacillus* or *Bifidobacterium.* 20 In addition to evaluation in the SENGSYN trial, we are liaising with the research team of the ongoing PROSYNK study in western Kenya (Pan Africa Clinical Trial Registry, PACTR202003893276712) to allow a comparison of the effects of the Labinic synbiotic in two different settings.

Despite the widespread and apparently safe use of probiotics in highly susceptible individuals, adverse effects of an administered *Lactobacillus* have been reported in sick children receiving intensive care.^21^ Although we are not recruiting sick children in this study, we will closely monitor SAEs.

We will start the intervention within the first 3 days of life to limit competition from other gut organisms that rapidly start to colonise the gut following birth. Administration will discontinue at 6 months as the introduction of complementary food is associated with a marked increase in diversity of the gut microbiota,^14^ likely reducing the effects of prebiotics, probiotics and synbiotics.

The same procedures that will be undertaken in all infants participating in the AASH cohorts, including the assessment of feeding and WASH practices, epigenetic status and characterisation of the gut microbiota, will also be undertaken in infants receiving the synbiotic in this study. This will provide a valuable opportunity to explore how synbiotic administration interacts with other domains and the mechanisms through which it may affect growth.

The SENGSYN study will establish an infrastructure in rural Senegal for evaluating alternative prebiotic, probiotic and synbiotic products and other potential approaches to modulate the gut microbiota in early life. This study will also further develop our understanding of the utility of the selected biomarkers in the evaluation of EED, which may allow us to test a series of novel interventions efficiently in advance of large-scale clinical trials.

## Supplementary Material

Author's manuscript

## Data Availability

Data sharing not applicable as no datasets generated and/or analysed for this study.
