# Supplementary material for: Improving gut health and growth in early life: a protocol for an individually randomised, two-arm, open-label, controlled trial of a synbiotic in infants in Kaffrine District, Senegal
Source: BMJ Paediatr Open. 2024 Feb 27;8(Suppl 1):e001629. doi: 10.1136/bmjpo-2022-001629 (PMC10900337; doi:10.1136/bmjpo-2022-001629)
Supplement: Supplementary data [file bmjpo-2022-001629supp001.pdf]

UKRI_GCRF_Stunting_Hub_SENGSYN_PIS_V2_0_of_12_June_2020

Addition to the GCRF Action Against Stunting Hub study; the Senegal Synbiotic Supplement (SENGSYN) study

Participant information sheet (PIS) for mothers/carers of newborns English versions

WORKSTREAM: **Gut Health; Senegal Synbiotic Supplement (SENGSYN) study**
VERSION & DATE: **Version 2.0 (12th June 2020)**
AUTHOR: **Stephen Allen; Babacar Faye; Doudou Sow**
APPROVED BY: **1) CNERS, Senegal; 2) LSTM REC, UK**
APPROVAL DATE: **1) November 11th 2020; 2) June 19th 2020**
WEB LOCATION FOR ETHICS: **1) https://www.cners.sn/ 2) https://www.lstmed.ac.uk/research/research-integrity/research-ethics-committee**

Workstream lead: Prof Stephen Allen; Room M-215, Department of Clinical Sciences, Liverpool School of Tropical Medicine, Pembroke Place, Liverpool L3 5QA, UK, Tel +44 (0)151 705 3752. Email: stephen.allen@lstmed.ac.uk

Principal Investigators:
Prof Faye Babacar (Lead Senegal); Dr Doudou Sow (Gut Health Lead Senegal)

<table>
<tr><td colspan="2">Revision chronology:</td><td>Signature Chief Investigator 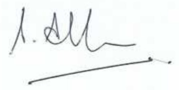</td></tr>
<tr><td>Participant Information Sheet (PIS) and consent statements (CS)</td><td>Version</td><td>Details of Changes</td></tr>
<tr><td>PIS/CS: Parents/carers</td><td>v1.0-13th April 2020</td><td>Original</td></tr>
<tr><td>PIS: Parents/carers</td><td>V2.0-12th June 2020</td><td>Responses to LSTM REC review (20-012), May 7th 2020 and the CNERS, Senegal REC review (SEN19/78) 23rd April 2020</td></tr>
<tr><td>CS: Parents/carers</td><td>V2.0-12th June 2020</td><td>Separate document</td></tr>
</table>

Momo Kadia B, *et al. BMJ Paediatrics Open* 2024; 8:e001629. doi: 10.1136/bmjpo-2022-001629

UKRI_GCRF_Stunting_Hub_SENGSYN_PIS_V2_0_of_12_June_2020

TABLE OF CONTENTS

1. Source documents in English used for subsequent translation 3
1.1. English 3
1.1.1. Participant Information Sheet for parents/carers (English) 3
1.1.2. Consent statement for screening and participation in the trial (English) 8

UKRI_GCRF_Stunting_Hub_SENGSYN_PIS_V2_0_of_12_June_2020

1. SOURCE DOCUMENTS IN ENGLISH USED FOR SUBSEQUENT TRANSLATION

1.1. ENGLISH

1.1.1. Participant Information Sheet for mothers/carers (English)

Addition to the GCRF Action Against Stunting Hub study;
the* Senegal Synbiotic Supplement (SENGSYN) *study

Participant Information Sheet: Mothers/carers of newborns

**Investigators**	
Prof Stephen Allen (PI)[1]	Prof Faye Babacar (Lead Senegal)[2]
Clinical Research Associate[1]: to be appointed	Assoc Prof. Doudou Sow (Gut Health lead; Senegal)[3]
**Investigators' institutions**	
1 Liverpool School of Tropical Medicine,	
2 Université Cheikh Anta DIOP, Dakar, Senegal	
3 Service de Parasitologie-Mycologie<br>Section Biologie et Explorations Fonctionnelles<br>UFR Sciences de la Santé, Senegal	

UKRI_GCRF_Stunting_Hub_SENGSYN_PIS_V2_0_of_12_June_2020

Addition to the GCRF Action Against Stunting Hub study; the Senegal Synbiotic Supplement (SENGSYN) *study*

Participant Information Sheet: Mothers/carers of newborns

Introduction

As you will know, you are already part of a study to find out why some otherwise healthy children do not grow as well as expected and become "stunted". One of the factors that contributes to poor growth is an unhealthy gut. An unhealthy gut means that the baby does not breakdown and absorb food (nutrients) as well as they should and is also more prone to infections. We call this gut problem "environmental enteric dysfunction". It is caused by the baby swallowing unhealthy bacteria (harmful microbes or germs) which damage the gut.

A very important way to protect the gut in young babies is exclusive breast feeding. This is where the baby only has breast milk and no other liquids or foods. We encourage all mothers to exclusively breast feed their babies. However, despite exclusive breast feeding and the best efforts of the mother and other carers to look after their baby, we know that many babies still get an unhealthy gut.

Beneficial bacteria live in a healthy gut and help fight-off harmful microbes. Babies acquire these beneficial bacteria naturally during a normal delivery and also during the first few months of life through breastfeeding. However, there are many factors that prevent young babies acquiring these beneficial bacteria in the gut.

In this study, we want to see if we can support the growth of beneficial bacteria in the gut in young babies. This is done in many countries and leads to health benefits. In a recent large study in India, boosting the beneficial bacteria in the gut of newborn babies prevented infections. This study will assess whether this approach benefits babies in Senegal.

We invite you and your baby to take part in this study. Before you decide whether to take part, it is important for you to understand why the study is being done and what it will involve. Please take the time to read the following information carefully. If you prefer, this information can be read to you. Please do ask us if there is anything that is not clear and if you would like more information. You can decide whether or not to take part up until your baby is four days old.

What is the purpose of the study?

In this study, we want to see whether we can boost the beneficial gut bacteria in babies to improve their growth. We will also test whether boosting the beneficial bacteria prevents infections and also improves gut health.

Why has my baby been chosen?

You have already been part of the Action Against Stunting study and have delivered a healthy baby. We will invite mothers/carers with a healthy baby to partake in this study. If you agree, we will check that your baby is OK to join the study.

What intervention is being tested?

The main types of beneficial bacteria that occur naturally in the gut in healthy, breastfed babies are Bifidobacteria and Lactobacilli. Many people take these bacteria as dietary supplements called "probiotics". Another approach is to give special sugars ("prebiotics") that support the growth of beneficial bacteria. The benefit may be greatest when a probiotic and a prebiotic are combined - called a "synbiotic". Prebiotics, probiotics and synbiotics have been tested in many research studies in many different countries, including in very small babies and sick people, and found to be safe.

In this study, we will give babies a dietary supplement of a synbiotic. This contains 3 different strains of Bifidobacteria and Lactobacilli that are present in products that are specially designed for babies and are already used in several countries. The label provided with the synbiotic contains more information. Please do ask if you would like to see this. These details are also provided at the end of this form.

The supplement is given as a small amount of powder that can be sprinkled directly into the baby's mouth before feeding or mixed in a clean container with expressed breast milk or sterile water.

After your baby has taken a feed well, the supplement will be given once a day for 10 days and then once weekly until your baby is 6 months old. Our research staff will either visit you at home or see you in a research clinic to help you to give the supplement.

We will compare the babies who receive the supplement with babies who do not get anything extra so we can see whether it improves growth. All babies in the study will receive special attention from the research team. This includes regular monitoring of growth and also support when babies are unwell.

What will happen if I want my baby to take part?
If you agree for your baby to join the study, your baby has a 1 in 3 chance of receiving the supplement. In other words, for every 3 babies that join the study, 1 will get the supplement and 2 will not receive anything extra. It is important that babies in the study do not take probiotic or synbiotic supplements other than those provided by the study team.

You have already agreed to provide information about feeding and how you keep the baby clean, for your baby to be measured regularly and provide samples of stool and blood. Apart from the extra visits, there will be no change; no extra samples or measurements are required for this study. The main activities of the study are summarised in the following diagram. A urine sample will also be collected at 6, 18 and 24 months as part of the main study.

UKRI_GCRF_Stunting_Hub_SENGSYN_PIS_V2_0_of_12_June_2020

Synbiotics in infants in Senegal; Overview

**Age of baby:**	**Birth**	**1m**	**2m**	**3m**	**4m**	**5m**	**6m**	**12m**	**18m**	**24m**
**Interventions:**	Arm 1	Synbiotic daily for 10 days then weekly								
	Arm 2	No supplement								
**Collection of information and samples**										
Weekly diary		√	√	√	√	√	√	√	√	√
Weight; Feeding	√	√	√	√	√	√	√	√	√	√
Measurements of length and head size		√		√			√	√	√	√
Stool sample		√					√			√
Blood sample							√			√

What will we do today?

If you agree for your baby to join the study, we will allocate the baby by chance to either receive the supplement or not. If your baby is allocated to the supplement, the staff will provide you with the first supplement and help you to give it to your baby. We will only start babies on a supplement once they have taken a feed well.

We will call you or send you telephone messages to remind you of the follow-up visits.

What else will be expected of me if I agree for my baby to be in the study?

If your baby is unwell at anytime during the study or if you think the supplement is causing any harmful effects, we would like you to contact us to let us know. You can do this by calling or texting one of the team or telling the staff at your health centre.

All information collected from you during the study will be used by staff and scientists involved in the study. This includes staff in Liverpool, UK. The information about you and your baby and the samples collected will be handled as explained in the information sheet for the main study. We will make sure that your information is kept private by removing your name and address. Your personal details will be destroyed within 10 years of the end of the study.

What are the possible disadvantages and risks of taking part?

The bacteria in the supplement in this study have been given to many babies without causing any harm. However, if you think the synbiotic causes any harmful effects in your baby, we will discuss with you if you need to stop giving it. If this happens, we would still like your baby to continue with

the follow-up. All babies will be given a thorough health check and any problems will be recorded. We will stop giving the supplement if we suspect that it is causing health problems.

The extra visits by our research staff and giving the supplement will be time consuming and may interfere with your normal daily schedule. However, we will keep the amount of time required to a minimum. Should the research staff identify any health conditions in your child during the study, they will notify you of this and help with referral for appropriate assessment.

Costs and compensation for being in the study
It is free to take part in the study. Although we do not expect that taking part in this study will incur any costs for you, you will receive reimbursement if you do incur costs that are required for your baby to take part in the study. These include travel costs (likely between £3-5) and inconvenience costs if required to attend a research clinic.

What if I don't want my baby to be in the study?
You are free to choose whether or not you want your baby to be part of this study and you have the right to refuse. If you decide that your baby should not join the study, he/she will continue in the Action Against Stunting Hub study and also receive all the standard health care that babies usually receive.

If you choose for your baby to participate in the study, you are free to change your mind and withdraw him/her from the study at any time. You do not have to give a reason. Even if you withdraw from the supplement study, your baby will still get the standard care that all babies receive.

Who is funding and sponsoring this study?
The study is funded by the United Kingdom Research and Innovation Global Challenges Research Fund. The sponsor responsible for the study is the Liverpool School of Tropical Medicine, Liverpool, UK

Contact information for questions or concerns
If you have any questions about this study, wish to make a complaint or if you want your child to stop being part of the study, please contact Porf Babacar Faye, Service de Parasitologie – Mycologie, Faculté de Médecine, Pharmacie et Odontologie, Université Cheikh Anta DIOP, Dakar BP : 5005 Dakar Fann / 16 949 Dakar Fann Tel: (221) 33 825 19 98; Email: bfaye67@yahoo.fr

You can also contact any of our study staff or staff at your health centre.

If you have any questions about you or your baby's rights as study participants, or if you think you or your child has been injured because of this study, please contact <<details of the Senegal REC>>
We hope the results of this study will help to improve growth and gut health in babies in this area.

Thank you very much for your time.

You will be given a copy of this information sheet and the signed consent form to keep for your records. One copy will be kept by the study staff.

UKRI_GCRF_Stunting_Hub_SENGSYN_PIS_V2_0_of_12_June_2020

Information about the synbiotic supplement: Labinic[*] Synbiotic.

This product has been manufactured for a clinical trial and should only be used according to the instructions in the study. It is completely free of dairy, lactose, gluten, colourings, stabilisers, preservatives, animal products and other allergens.

INSTRUCTIONS FOR USE

Before using this product, read and keep this leaflet. If you notice any side-effects, then please tell your health professional straight away. This is an edible, dietary supplement. It is not a medicine. It is manufactured to high standards. Each batch is monitored for contaminants. This product is designed for use in SENGSYN study and should be used according to the study instructions.

Contents

Labinic Synbiotic is presented in strips of blister-packed white/white capsules in a poly-foil bag with silica sachets. Each capsule contains a powder containing:
(Active ingredients) 5 billion (total) of *Lactobacillus acidophilus* NCFM, *Bifidobacterium bifidum* Bb-06 and *Bifidobacterium infantis* Bi-26 probiotics and BENEO Orafti® Synergy1[**]; 50 oligofructose:50 FOS; 200mg; (Inactive ingredients Ph102 microcrystalline cellulose 280mg, Magnesium stearate 10mg, Aerosil 200 Pharma 5mg).

How to take/administer Labinic Synbiotic

Remove capsule from blister pack just before use. Open the capsule and mix contents with a small amount of breastmilk in a clean container (or sterile water if baby is not breast fed) or sprinkle directly into the baby's open mouth before a feed.

Labinic Synbiotic - Possible side-effects

Dietary supplements containing live enteric bacteria are usually well tolerated. Labinic Synbiotic may occasionally cause abdominal discomfort, diarrhoea and/or flatulence. Not recommended for use where gut or general immunity is compromised. Systemic infection is possible but very rare. Do not use Labinic Synbiotic if recipient is allergic or hypersensitive to any of the ingredients. Seek urgent treatment from a doctor or a member of the research team if taking Labinic Synbiotic makes recipient feel ill or there are any concerns about rashes or infection. Responsibility for safety and monitoring rests with the research team.

Do not exceed the recommended dose. There are no reports of adverse effects from the use or overdose of these probiotics at the time of production of this information leaflet.

Storage

We recommend keeping the blister-pack in the poly-foil bag which should be stored in a cool place away from direct heat or sunlight.
Do not use after expiry date (see packaging).

KEEP OUT OF THE REACH AND SIGHT OF CHILDREN

www.biofloratech.com

[*]Labinic is a registered trademark of Biofloratech Ltd, Image Court 139, 328 - 334 Molesey Rd, Walton-on-Thames, Surrey KT12 3LT, United Kingdom
[**]Beneo Orafti® Synergy 1 is a registered trademark of BENEO GmbH, Maximilianstrasse 10, 68165 Mannheim, Germany
