# Supplementary material for: Improving gut health and growth in early life: a protocol for an individually randomised, two-arm, open-label, controlled trial of a synbiotic in infants in Kaffrine District, Senegal
Source: BMJ Paediatr Open. 2024 Feb 27;8(Suppl 1):e001629. doi: 10.1136/bmjpo-2022-001629 (PMC10900337; doi:10.1136/bmjpo-2022-001629)
Supplement: Supplementary data [file bmjpo-2022-001629supp002.pdf]

*UKRI_GCRF_Stuning_Hub_SENGSYN_Consent_form_V_2_0_of_12_June_2020*

# Addition to the GCRF Action Against Stunting Hub study; the Senegal Synbiotic Supplement (SENGSYN) study

## Consent statement for mothers/carers of newborns English versions

This statement refers to the Action Against Stunting Hub SENGSYN study Participant information sheet (PIS) for mothers/carers of newborns (v2.0-12th June 2020)

| Consent Statement for Eligibility assessment and Participation in the study | |
|---|---|
| Your signature below means that you voluntarily agree for your baby to participate in this research | |
| **Assessment for eligibility for inclusion** | Please initial each box: |
| The above study has been explained to me and I have been given the opportunity to ask questions. I agree for my baby to be assessed to see if he/she is suitable to be in the study. | |
| I understand that my baby's further participation in the study will depend on the results of the eligibility assessment. I understand that my agreeing for my baby to be assessed does not mean that I have to agree for my baby to be in the study. | |
| I understand that I can change my mind at any time without having to give a reason and without any consequences to my baby's health care. | |
| **Participation in the SENGSYN study** | |
| I understand that I am free to choose whether or not my baby will join this study. I understand that saying "No" will allow my baby to continue in the Action Against Stunting study and not effect the usual care that my baby receives. | |
| I understand that there will be no change to the questionnaires, measurements and sample collection that I have already agreed to for the Action Against Stunting Hub study. | |
| I understand that the study team may make extra visits to me at home and in research clinics. I agree for the study team to contact me by phone and to receive mobile phone messages to remind me of the study visits. | |
| I have read this form and decided that I agree for my baby to take part in the study including taking the synbiotic supplement if required. The study's general objectives, details of participation and possible risks and inconveniences have been explained to me to my satisfaction. I understand that I may withdraw my consent at any time. My signature also indicates that I have received a copy of this consent form. | |

*UKRI_GCRF_Stuning_Hub_SENGSYN_Consent_form_V_2_0_of_12_June_2020*

| ID number: _._._ _ _ | **Name** | **Signature** | **Today's date** |
|---|---|---|---|
| Adult providing consent for baby | | | |
| Relationship to baby | Mother / other carer: specify ………………..…………………..………………. | | |
| Witness* | | | |
| Study staff consenting participant | | | |
| * A witness is only needed if the participant cannot read or sign the consent form. The witness must be a person independent from the study. The participant must verbally state that he/she has had the information sheet read and explained to them and they give their consent for their baby to participate in the study in the presence of a witness who will then sign. | | | |
