# Supplementary material for: Improving gut health and growth in early life: a protocol for an individually randomised, two-arm, open-label, controlled trial of a synbiotic in infants in Kaffrine District, Senegal
Source: BMJ Paediatr Open. 2024 Feb 27;8(Suppl 1):e001629. doi: 10.1136/bmjpo-2022-001629 (PMC10900337; doi:10.1136/bmjpo-2022-001629)
Supplement: Author's manuscript [file bmjpo-2022-001629.draft_revisions.pdf]

BMJ Paediatrics Open

BMJ Paediatrics Open is committed to open peer review. As part of this commitment we make the peer review history of every article we publish publicly available.

When an article is published we post the peer reviewers' comments and the authors' responses online. We also post the versions of the paper that were used during peer review. These are the versions that the peer review comments apply to.

The versions of the paper that follow are the versions that were submitted during the peer review process. They are not the versions of record or the final published versions. They should not be cited or distributed as the published version of this manuscript.

BMJ Paediatrics Open is an open access journal and the full, final, typeset and author-corrected version of record of the manuscript is available on our site with no access controls, subscription charges or pay-per-view fees (http://bmjpaedsopen.bmj.com).

If you have any questions on BMJ Paediatrics Open's open peer review process please email info.bmjpo@bmj.com

BMJ Paediatrics Open

Improving gut health and growth in early life: a protocol for an individually randomised, two-arm, open-label, controlled trial of a synbiotic in infants in Kaffrine District, Senegal

Journal:	*BMJ Paediatrics Open*
Manuscript ID	bmjpo-2022-001629
Article Type:	Protocol
Date Submitted by the Author:	02-Aug-2022
Complete List of Authors:	Momo Kadia, Benjamin; Liverpool School of Tropical Medicine Department of Clinical Sciences, Department of Clinical Sciences<br>Khouma, Marietou; Cheikh Anta Diop University of Dakar<br>Sow, Doudou; Université Gaston Berger<br>Faye, Babacar; Cheikh Anta Diop University of Dakar<br>Ramsteijn, Anouschka S.; University of Aberdeen<br>Calvo-Urbano, Beatriz; University of London, Department of Pathobiology and Population Sciences<br>Jobarteh, Modou; London School of Hygiene & Tropical Medicine<br>Ferguson, Elaine; London School of Hygiene & Tropical Medicine<br>Haggarty, Paul; University of Aberdeen<br>Webster, Joanne P.; University of London, Department of Pathobiology and Population Sciences<br>Walker, Alan W.; University of Aberdeen<br>Heffernan, Claire; London School of Hygiene & Tropical Medicine; London International Development Centre<br>Allen, Stephen; Liverpool School of Tropical Medicine, Department of Clinical Sciences
Keywords:	Growth, Gastroenterology, Microbiology, Parasitology, Molecular Biology

SCHOLARONE™
Manuscripts

BMJ

I, the Submitting Author has the right to grant and does grant on behalf of all authors of the Work (as defined in the below author licence), an exclusive licence and/or a non-exclusive licence for contributions from authors who are: i) UK Crown employees; ii) where BMJ has agreed a CC-BY licence shall apply, and/or iii) in accordance with the terms applicable for US Federal Government officers or employees acting as part of their official duties; on a worldwide, perpetual, irrevocable, royalty-free basis to BMJ Publishing Group Ltd ("BMJ") its licensees and where the relevant Journal is co-owned by BMJ to the co-owners of the Journal, to publish the Work in this journal and any other BMJ products and to exploit all rights, as set out in our licence.

The Submitting Author accepts and understands that any supply made under these terms is made by BMJ to the Submitting Author unless you are acting as an employee on behalf of your employer or a postgraduate student of an affiliated institution which is paying any applicable article publishing charge ("APC") for Open Access articles. Where the Submitting Author wishes to make the Work available on an Open Access basis (and intends to pay the relevant APC), the terms of reuse of such Open Access shall be governed by a Creative Commons licence – details of these licences and which Creative Commons licence will apply to this Work are set out in our licence referred to above.

Other than as permitted in any relevant BMJ Author's Self Archiving Policies, I confirm this Work has not been accepted for publication elsewhere, is not being considered for publication elsewhere and does not duplicate material already published. I confirm all authors consent to publication of this Work and authorise the granting of this licence.

Confidential: For Review Only

Improving gut health and growth in early life: a protocol for an individually randomised, two-arm, open-label, controlled trial of a synbiotic in infants in Kaffrine District, Senegal

Authors:

Benjamin Momo Kadia[1], Marietou Khouma[2], Doudou Sow[3], Babacar Faye[2], Anouschka S. Ramsteijn[4], Beatriz Calvo-Urbano[5], Modou L. Jobarteh[6], Elaine Ferguson[6], Paul Haggarty[4], Joanne P. Webster[5], Alan W. Walker[4], Claire Heffernan[5,6,7], Stephen J Allen[1]

Authors' affiliations

1. Department of Clinical Sciences, Liverpool School of Tropical Medicine (LSTM), Liverpool, United Kingdom (UK)
2. Service de Parasitologie-Mycologie, Faculté de Médecine, Université Cheikh Anta Diop (UCAD), Dakar, Senegal
3. Service de Parasitologie-Mycologie, UFR Sciences de la Santé, Université Gaston Berger, Saint Louis, Sénégal
4. Rowett Institute, University of Aberdeen, Aberdeen, UK
5. Department of Pathobiology and Population Sciences, Royal Veterinary College, University of London, Herts, AL9 7TA, UK
6. London School of Hygiene and Tropical Medicine (LSHTM), London, UK
7. London International Development Centre (LIDC), London, UK

Authors' emails

BMK: benjamin.kadia@lstmed.ac.uk
MK: khoumarita93@gmail.com
DS: doudou.sow@ugb.edu.sn
BF: babacar2.faye@ucad.edu.sn
ASR: anouschka.ramsteijn@abdn.ac.uk
BCU: bcalvo@rvc.ac.uk
MLJ: modou.jobarteh@lshtm.ac.uk
EF: elaine.ferguson@lshtm.ac.uk
PH: p.haggarty@abdn.ac.uk
JPW: jowebster@rvc.ac.uk
AWW: alan.walker@abdn.ac.uk
CH: Claire.Heffernan@lidc.ac.uk
SA: stephen.allen@lstmed.ac.uk

Corresponding author

Benjamin Momo Kadia; LSTM; email: benjamin.kadia@lstmed.ac.uk

Confidential: For Review Only

Abstract

Introduction: Infants exposed to enteropathogens through poor sanitation and hygiene can develop a subclinical disorder of the gut called environmental enteric dysfunction (EED), characterised by abnormal intestinal histology and permeability. EED can contribute to stunting through reduced digestion and absorption of nutrients, increased susceptibility to infections, increased systemic inflammation and inhibition of growth hormones. EED can be apparent by age 12 weeks, highlighting the need for early intervention. Modulating the early life gut microbiota using synbiotics may improve resistance against colonisation of the gut by enteropathogens, reduce EED and improve linear growth.

Methods and analysis: An individually randomised, two-arm, open-label, controlled trial will be conducted in Kaffrine District, Senegal. Infants will be recruited at birth and randomised to either receive a synbiotic containing two *Bifidobacterium* strains and one *Lactobacillus* strain, or no intervention, during the first 6 months of life. The impact of the intervention will be evaluated primarily by comparing length-for-age z score at 12 months of age in infants in the intervention and control arms of the trial. Secondary outcome variables include biomarkers of intestinal inflammation, intestinal integrity and permeability, gut microbiota profiles, presence of enteropathogens, systemic inflammation, growth hormones, epigenetic status, and episodes of illness during follow-up to age 24 months.

Discussion: This trial will contribute to the evidence base on the use of a synbiotic to improve linear growth by preventing or ameliorating EED in a low-resource setting.

Ethics and Dissemination: This trial has been approved by the National Ethics Committee for Scientific Research in Senegal and the Ethics Committees of Liverpool School of Tropical Medicine (LSTM) and London School of Hygiene and Tropical Medicine (LSHTM). Key stakeholders will be informed of the findings and the results submitted for presentation at national and international conferences and for publication in peer-reviewed journals.

Trial Registration: Pan African Clinical Trials Registry; PACTR202102689928613 (https://pactr.samrc.ac.za/TrialDisplay.aspx?TrialID=14491)

Confidential: For Review Only

Summary box

What is known about the subject?

- Environmental enteric dysfunction (EED) reduces the digestion and absorption of nutrients, and increases susceptibility to infections and systemic inflammation, contributing to growth faltering in children.
- EED can appear as early as 12 weeks of age, and measures including exclusive breastfeeding, nutritional supplements and improved water, sanitation and hygiene have not been sufficient to prevent EED and subsequent growth faltering in at-risk populations.
- Supplementation with a synbiotic may enhance colonisation resistance against enteropathogens and improve gut health.

What this study hopes to add:

- Evidence of whether administration of a synbiotic in early life improves linear growth in infants in a community exposed to poor hygiene and sanitation.
- Evidence of the effect of a synbiotic on biomarkers of gut health, enteropathogen colonisation, systemic inflammation, epigenetic status, and growth.

Confidential: For Review Only

Introduction

Globally, stunting affects about 149.2 million children under five years of age, with 40% of affected children residing in Africa [1]. Stunting is a complex process that manifests physically as significantly impaired linear growth, defined by a length-for-age/height-for-age z-score more than two standard deviations below the World Health Organization Child Growth Standards median [2,3].

Recent large cluster randomised trials in Bangladesh [4], Kenya [5] and Zimbabwe [6] reported a limited impact of water, sanitation, and hygiene (WASH) interventions and provision of food supplements on linear growth. Several studies have, however, demonstrated that growth faltering is associated with an enteropathy termed environmental enteric dysfunction (EED) [7, 8]. EED is a subclinical disorder that results from damage to the intestinal mucosa by pathogenic microbes [7,8]. It is characterised by villous atrophy, intestinal inflammation, and a 'leaky' intestinal mucosa [9]. The consequences are reduced nutrient digestion and absorption, and systemic inflammation that reduces the production of growth hormones [10,11]. EED can occur as early as age 12 weeks, and despite exclusive breastfeeding [10]. Biomarkers in stool and blood have been proposed as non-invasive and accessible indices of EED [9].

One potential target for novel interventions to prevent or reduce stunting is the diverse and numerous collections of microbes ('microbiota') that colonises the gut of infants. The gut microbiota is critical for the development of the gut and other organs, mucosal and systemic immunity, and protection against gastrointestinal infections through a process termed 'colonisation resistance' [12–14]. However, the development of the gut microbiota in early life can be perturbed by factors such as caesarean section delivery and exposure to pathogens and antibiotics [15].

Synbiotics, which are a combination of (a) prebiotic(s) and (b) probiotic(s), are one type of intervention with microbiota-modulating potential. Prebiotics are substrates that selectively promote the growth and activity of beneficial host microorganisms, thereby conferring a health benefit [16]. Probiotics are live microorganisms which, when administered in adequate amounts, can confer health benefits to the host [17]. In a large trial in India, a synbiotic given for just seven days in newborns, nearly all of whom were exclusively breastfed, significantly reduced sepsis, pneumonia, and skin infections [18]. This landmark study showed that synbiotic administration is acceptable to mothers, feasible at scale and may have important health benefits. However, the study did not report effects on gut health or linear growth.

The primary objective of this trial is to determine whether supplementation with a synbiotic during the first six months of life improves linear growth of children at age 12 months. The trial will also assess the effects of the synbiotic on biomarkers of intestinal inflammation and integrity, systemic inflammation, gut microbiota maturation, levels of growth hormones, gut colonisation with enteropathogens, host epigenetic status relevant to gut and general health and growth, episodes of illness, and linear growth up to age 24 months.

Confidential: For Review Only

Methods and analysis

Trial design

SENeGal SYNbiotic (SENGSYN) is an individually randomised, two-arm, open-label, controlled trial. The protocol for this trial has been developed in accordance with the Standard Protocol Items: Recommendations for Interventional Trials (SPIRIT) guideline. The trial is part of the research conducted within the UK Research and Innovation Global Challenges Research Fund (UKRI GCRF) AASH (Action Against Stunting Hub) (https://actionagainststunting.org/) – an interdisciplinary research consortium investigating the precursors of child stunting to inform programmes and policies to reduce the global burden of this condition. Infants born to women recruited during pregnancy in Kaffrine, Senegal in the AASH observational study will be assessed for eligibility and recruited to participate in the SENGSYN trial.

Recruited infants will be randomised to receive either a daily supplement of a synbiotic in the first ten days of enrolment and then weekly up to 6 months of age, or no intervention, with the children followed up to 24 months of age. Detailed anthropometry will be conducted at birth and months 3, 6, 12, 18 and 24, stool samples will be collected at 1, 6 and 24 months, and blood samples will be collected at 6 and 24 months. Assessments of morbidity and breastfeeding are conducted weekly up to 24 months of age (Table 1).

https://mc.manuscriptcentral.com/bmjpo

Table 1: Table of the schedule of enrolment, intervention, and assessments. The trial follows the SPIRIT (Standard Protocol Items: Recommendations for Interventional Trials) protocol. m=months

	STUDY PERIOD										
	**Enrolment/ Allocation**	**Post-allocation**									**Close-out**
**TIMEPOINT**	Birth	1m	2m	3m	4m	5m	6m	9m	12m	18m	24m
**ENROLMENT**											
**Eligibility screen**	X										
**Informed consent**	X										
**Random allocation**	X										
**INTERVENTION**											
**Intervention arm**		Synbiotic daily for 10 days then weekly till 6 months									
**Control arm**		Routine care									
**ASSESSMENTS**											
**Weekly morbidity diary**		X	X	X	X	X	X	X	X	X	X
**Body weight, breastfeeding**	X	X	X	X	X	X	X	X	X	X	X
**Other anthropometry**		X		X			X	X	X	X	X
**Stool pathogens/ microbiota/biomarkers**		X					X				X
**Blood biomarkers**							X				X
**Epigenetic status in saliva**		X									X

Study setting

The trial is based in Kaffrine District, Senegal. The district has 28 health posts or clinics and 32 health huts administered by the Kaffrine Health Centre. The AASH will recruit participants in seven clinics, and women participating in the AASH study will be targeted for the recruitment of their newborns in the SENGSYN study.

Participants

Pregnant women recruited into the AASH observational study will be given an information sheet about this synbiotic trial at recruitment (supplemental file 1). The women will be further approached by study staff at health facilities or in their homes within 3 days of delivery to determine whether their newborn meets the inclusion criteria and if she/he does, to obtain informed consent for participating in the SENGSYN trial.

Inclusion criteria are:

- Singleton newborn
- Birthweight (BW) or current weight (if BW not known) ≥2000 g
- Healthy infant who is breastfed and has taken at least one breastfeed well
- Age 1-3 days

Confidential: For Review Only

Exclusion criteria are:

- Multiple birth (e.g., twins, triplets, etc.)
- Presence of any acute illness in the newborn (e.g., fever, receiving treatment with antibiotics)
- Congenital abnormality that might be life-threatening or impair growth
- Infant with potential contraindication to synbiotic (e.g., suspected immune suppression, cardiac abnormality)
- Mother unlikely to stay in study area for the duration of the trial
- Any health or study staff concerns regarding safety to participate in the trial

Infants of women known to be HIV positive and without known immunosuppression are eligible to participate in the trial.

Randomisation and allocation

The trial statistician will prepare a computer-generated random allocation sequence using R-coding, with blocks of random size stratified by clinic, allocating newborns to one of two study arms:

Arm 1: Labinic synbiotic daily for 10 days and then weekly to age 6 months
Arm 2: Control group; usual care with no supplement

Participants (infants) will be allocated at a ratio of 1:2 in the intervention and control arms respectively. The allocation sequence will be held by an independent pharmacist in Senegal who will prepare sequentially numbered, sealed, opaque envelopes according to the random allocation sequence. The random allocation sequence will be concealed from all other members of the research team including staff allocating newborns to the trial arms and laboratory staff analysing the biological samples. Each envelope will be opened sequentially following recruitment of each newborn and will contain a card indicating the trial arm. The trial arm will then be linked to the infant's unique AASH identification number. Mothers/carers who decline for their infant to participate in the SENGSYN study will continue in the AASH observational cohort.

Following participant allocation, study staff will assist the mother/carer in administering the first dose of the synbiotic to infants in the intervention arm.

Intervention

The trial will explore the impact of the Labinic synbiotic, composed of a prebiotic (BENEO Orafti Synergy1; 50% oligofructose/50% fructooligosaccharide; 200 mg) plus three live bacterial strains (*Lactobacillus acidophilus* NCFM, *Bifidobacterium infantis* Bi-26 and *Bifidobacterium bifidum* Bb-06; total of 5 billion organisms/dose (Biofloratech Ltd, Walton-on-Thames, Surrey, UK). The synbiotic supplement is a powder in capsules with one dose per capsule. The contents of the capsule can be sprinkled directly into the infant's open mouth before feeding. Study staff will visit daily for 9 days after the initial dose and then weekly to age 6 months (32 doses in total) to supervise the administration of the synbiotic and record adherence. Administration will be repeated once if the infant vomits within 30 minutes of synbiotic administration. In infants taking antibiotics, including HIV-exposed infants taking daily co-trimoxazole, the synbiotic will be administered where possible at least 4 hours before/after the antibiotic or between doses to minimise the effect of the antibiotic on the synbiotic microbes. Antibiotic usage will be recorded during dosing visits and morbidity follow-up. Infants randomised to the control arm will receive the same follow-up as infants in the

https://mc.manuscriptcentral.com/bmjpo

AASH observation cohort. All infants will receive routine care as per national guidelines and will have contact details and mobile phone numbers of study staff for assistance in case of infant illness.

Outcomes

All infants will be followed up to 24 months of age with anthropometry, biological sample collections (blood, stool and saliva), and morbidity assessment conducted at scheduled time points (Table 1).

Primary outcome

The primary outcome of the trial is linear growth at age 12 months assessed by length-for-age z score. Regularly calibrated equipment for anthropometry will include a Model 876 SECA/infant scale, UNICEF stadiometer/infantometer, Lufkin WP601 measuring tape and Holtain skinfold caliper. Measurements will be conducted in duplicate with a third measurement taken if the first two measurements do not agree within a specified amount. Weight will be recorded to the nearest 100 g and height, length, mid upper arm circumference (MUAC), head circumference, triceps and sub-scapular skinfolds, and knee-to- heel length to the nearest 0.1 cm. Measurements will be used to determine z-scores using the WHO growth standards for length-for-age (LAZ), weight-for-length (WLZ), triceps-for-age and sub-scapular-for-age. Children will be classified as stunted or wasted if their LAZ or WLZ is <-2SD, respectively.

Secondary outcomes

The secondary outcomes are impact of synbiotic consumption on the presence of enteropathogens and parasites in stools, biomarkers of gut health in stool and blood, biomarkers of systemic inflammation, growth hormones, faecal microbiota composition, and epigenetics markers. Stool samples collected at 1, 6 and 24 months will be analysed using multiple methods including bacterial culture, qPCR (quantitative polymerase chain reaction) and ELISA (enzyme linked immunosorbent assay) for the following:

a) Enteropathogens: *Salmonella, Shigella,* pathogenic *Escherichia coli*
b) Enteroparasites: helminths (*Ascaris lumbricoides*, *Ancylostoma duodenale*, *Necator americanus*, *Trichuris trichiura*, *Strongyloides stercoralis)*, and protozoans (*Giardia lamblia*, *Cryptosporidia parvum/hominis* and *Entamoeba histolytica/dispar)*
c) Intestinal inflammation: myeloperoxidase
d) Intestinal permeability: α_1-antitrypsin
e) Maturation of gut microbiota, assessed using Illumina-based 16S rRNA gene amplicon sequencing

Blood samples collected at 6 and 24 months will be analysed for:

a) Chronic inflammation: alpha-1-acid glycoprotein (AGP)
b) Acute inflammation: C-reactive protein
c) Gut mucosal integrity: intestinal fatty acid binding protein
d) Growth hormones: insulin-like growth factor (IGF)-1 and its carrier protein IGF binding protein 3

Saliva samples collected within one month of birth and at 18-24 months will be analysed for epigenetic status including markers of gut and general health and growth.

Confidential: For Review Only

Sample size determination

The sample size calculation is based on observing a clinically meaningful improvement in linear growth in the intervention arm. In the WASH Benefits study in Kenya, the control group (n=1,431) at median age 12 months (range 2-18 months) had a mean length-for-age z score (LAZ) of -1.13 (SD=1.13)[5]. To observe a 25% improvement in LAZ at age 12 months (i.e., LAZ = -0.8475; SD=1.13) with 80% power, α =0.05 and with 1:2 group allocation, would require 189 infants in the intervention group and 378 in the control group. We plan to allow for 20% loss to follow-up by recruiting 236 to the intervention group and 472 to the control arm. Based on previous studies, we consider that this number of infants will also be sufficient to observe meaningful differences in biomarkers between the study arms. [8,10,12,19].

Data management and statistical analysis

Data and sample management and governance

Data and sample management will follow procedures established by the AASH. Data will be transmitted via secure file transfer protocols between Senegal and hub partners in the UK, Indonesia and India. A data monitoring and evaluation committee, a trial steering committee, research ethics committees of LSTM, and LSHTM, and the national ethics committee for scientific research in Senegal will oversee the conduct of the trial. The trial sponsor, LSTM, will review the trial at initiation, during the study and at trial closeout with additional monitoring visits as required.

Statistical analysis

Analysis of the primary and secondary endpoints will be based on intention to treat. Mean/median values of biomarkers, the proportion of infants with abnormal biomarker concentrations, gut colonisation with enteropathogens and the proportion of infants/children with episodes of illnesses will be compared in the two arms at 1, 6 and 24 months. Additional analysis of the primary and secondary endpoints will also be presented according to adherence to synbiotic administration. Data will be analysed using generalised linear models with treatment as the only predictor, generating the estimates of treatment effects and their 95% confidence intervals. Adjustment for co-variates and inclusion of interaction terms will be performed as appropriate. More details on statistical analysis methods using secondary variables are described in other papers in the supplement.

Patient and public involvement

There was no involvement of patients or the public in the design of this trial.

Protocol version

Version 2.0 of 12 June 2020

Discussion and conclusion

Childhood stunting affects many children in vulnerable populations, particularly in Africa, while interventions targeting WASH and nutrition have had limited success improving linear growth. Evidence has accumulated for suboptimal gut health and EED contributing to early life growth faltering. This clinical trial aims at evaluating the effectiveness of a synbiotic in improving linear growth in early life, as a potential affordable and scalable public health intervention.

Confidential: For Review Only

There are many potential pre-, pro- and synbiotic preparations on the market, with different mixtures of substrates and microorganisms. However, the limited knowledge of their effects on the gut complicates the selection of specific products for evaluation in clinical trials, including for the prevention or amelioration of EED. Identifying probiotics with specific anti-pathogen properties relevant to EED is difficult given the limitations of *in vitro* and animal models in mimicking the complex environment of the infant's gut, and the numerous enteropathogens that may contribute to EED [10]. Colonisation resistance is mediated through multiple mechanisms, and some are likely shared amongst *Lactobacillus* and *Bifidobacterium* species, including production of fermentation acids and acidifying the gut lumen [15]. Probiotics may have additional beneficial effects in EED through reducing intestinal inflammation and permeability [20]. Prebiotics may supplement human milk oligosaccharides, the natural prebiotics in breast milk, with additional carbon sources to promote the growth of putatively beneficial microbes such as bifidobacteria [21]. Prebiotics may also serve as decoy receptors for enteropathogens [22].

Lactobacilli and bifidobacteria are supplied routinely to about 70% of preterm infants nursed in neonatal units in Germany [23], based on their potential to prevent necrotising enterocolitis [18] and late onset sepsis [5]. A recent survey reported that Labinic was used routinely in preterm infants in seven neonatal units in the UK [24]. The Labinic probiotic has been associated with a significant decrease in necrotising enterocolitis and late onset sepsis in preterm infants with no episodes of sepsis due to *Lactobacillus* or *Bifidobacterium* [19]. In addition to evaluation in the SENGSYN trial, we are liaising with the research team of the on-going PROSYNK study in western Kenya (Pan Africa Clinical Trial Registry; PACTR202003893276712) to allow a comparison of the effects of the Labinic synbiotic in two different settings.

Despite the widespread and apparently safe use of probiotics in highly susceptible individuals, adverse effects of an administered *Lactobacillus* have been reported in sick children receiving intensive care [20]. Although we are not recruiting sick children in this study, we will closely monitor serious adverse events.

We will start the intervention within the first 3 days of life to limit competition from other gut organisms that rapidly start to colonise the gut following birth. Administration will discontinue at 6 months as the introduction of complementary food is associated with a marked increase in diversity of the gut microbiota [14] likely reducing the effects of pre-, pro- and synbiotics.

The same procedures that will be undertaken in all infants participating in the AASH cohorts, including the assessment of feeding and WASH practices, epigenetic status, and characterisation of the gut microbiota, will also be undertaken in infants receiving the synbiotic in this study. This will provide a valuable opportunity to explore how synbiotic administration interacts with other domains and the mechanisms through which it may affect growth.

The SENGSYN study will establish an infrastructure in rural Senegal for evaluating alternative pre-, pro- and synbiotic products and other potential approaches to modulate the gut microbiota in early life. This study will also further develop our understanding of the utility of the selected biomarkers in the evaluation of EED, which may allow us to test a series of novel interventions efficiently in advance of large-scale clinical trials.

Confidential: For Review Only

References

1. World Health Organization, World Bank, United Nations International Children's Emergency Fund. Levels and trends in child malnutrition: Key findings of the 2020 Edition of the Joint Child Malnutrition Estimates. Available at https://www.who.int/publications-detail-redirect/9789240025257

2. Prendergast AJ, Humphrey JH. The stunting syndrome in developing countries. Paediatr Int Child Health. 2014;34:250–65. doi.10.1179/2046905514Y.0000000158

3. de Onis M, Branca F. Childhood stunting: A global perspective. Matern Child Nutr. 2016;12:12–26. doi.10.1111/mcn.12231

4. Budge S, Parker AH, Hutchings PT, Garbutt C. Environmental enteric dysfunction and child stunting. Nutr Rev. 2019;77:240–253. doi.10.1093/nutrit/nuy068

5. Null C, Stewart CP, Pickering AJ, et al. Effects of water quality, sanitation, handwashing, and nutritional interventions on diarrhoea and child growth in rural Kenya: a cluster-randomised controlled trial. Lancet Glob Heal. 2018;6:e316–29. doi.org/10.1016/S2214-109X(18)30005-6

6. Luby SP, Rahman M, Arnold BF, et al. Effects of water quality, sanitation, handwashing, and nutritional interventions on diarrhoea and child growth in rural Bangladesh: a cluster randomised controlled trial. Lancet Glob Heal. 2018;6:e302–15. doi.org/10.1016/S2214-109X(17)30490-4

7. Kosek MN, Ahmed T, Bhutta ZA, et al. Causal Pathways from Enteropathogens to Environmental Enteropathy: Findings from the MAL-ED Birth Cohort Study. EBioMedicine. 2017;18:109–117. doi.10.1016/j.ebiom.2017.02.024

8. George CM, Burrowes V, Perin J, et al. Enteric Infections in Young Children are Associated with Environmental Enteropathy and Impaired Growth. Trop Med Int Heal. 2018;23:26–33. doi.10.1111/tmi.13002

9. Tickell KD, Atlas HE, Walson JL. Environmental enteric dysfunction: A review of potential mechanisms, consequences and management strategies. BMC Med. 2019;17:1–9. doi.10.1186/s12916-019-1417-3

10. Naylor C, Lu M, Haque R, et al. Environmental Enteropathy, Oral Vaccine Failure and Growth Faltering in Infants in Bangladesh. EBioMedicine. 2015;2:1759–1766. doi.10.1016/j.ebiom.2015.09.036

11. Prendergast AJ, Kelly P. Interactions between intestinal pathogens, enteropathy and malnutrition in developing countries. Curr Opin Infect Dis. 2016;29:229–36. doi.10.1097/QCO.0000000000000261

12. Arrieta MC, Stiemsma LT, Amenyogbe N, Brown E, Finlay B. The intestinal microbiome in early life: Health and disease. Front Immunol. 2014;5:47. doi.10.3389/fimmu.2014.00427

13. Buffie CG, Pamer EG. Microbiota-mediated colonization resistance against intestinal pathogens.

Confidential: For Review Only

Nat Rev Immunol. 2013;13:790–801. doi.10.1038/nri3535

14. Shanahan F, Van Sinderen D, O'Toole PW, Stanton C. Feeding the microbiota: Transducer of nutrient signals for the host. Gut. 2017;66:1709–17. doi.10.1136/gutjnl-2017-313872

15. Vandenplas Y, Carnielli VP, Ksiazyk J, et al. Factors affecting early-life intestinal microbiota development. Nutrition. 2020;78:110812. doi.10.1016/j.nut.2020.110812

16. Gibson GR, Hutkins R, Sanders ME, et al. Expert consensus document: The International Scientific Association for Probiotics and Prebiotics (ISAPP) consensus statement on the definition and scope of prebiotics. Nat Rev Gastroenterol Hepatol. 2017;14:491–502. doi.10.1038/nrgastro.2017.75

17. Food and Agriculture Organization (FAO)/World Health Organization (WHO). Guidelines for the Evaluation of Probiotics in Food. London, Ontario, Canada; 2002. Available from: http://www.who.int/foodsafety/publications/fs_management/probiotics2/en/

18. Panigrahi P, Parida S, Nanda NC, Satpathy R, Pradhan L, Chandel DiS, et al. A randomized synbiotic trial to prevent sepsis among infants in rural India. Nature. 2017;548:407–12. doi.10.1038/nature23480

19. Robertson C, Savva GM, Clapuci R, Jones J, Maimouni H, Brown E, et al. Incidence of necrotising enterocolitis before and after introducing routine prophylactic Lactobacillus and Bifidobacterium probiotics. Arch Dis Child Fetal Neonatal Ed. 2020;105:380–6. doi.10.1136/archdischild-2019-317346

20. Yelin I, Flett KB, Merakou C, et al. Genomic and epidemiological evidence of bacterial transmission from probiotic capsule to blood in ICU patients. Nat Med. 2019;25:1728–32. Available from: 10.1038/s41591-019-0626-9

21. Lawson, M.A.E., O'Neill, I.J., Kujawska, M. *et al.* Breast milk-derived human milk oligosaccharides promote *Bifidobacterium* interactions within a single ecosystem. *ISME J* **14,** 635–648 (2020). https://doi.org/10.1038/s41396-019-0553-2

22. Monteagudo-Mera A, Rastall RA, Gibson GR, Charalampopoulos D, Chatzifragkou A. Adhesion mechanisms mediated by probiotics and prebiotics and their potential impact on human health. Appl Microbiol Biotechnol. 2019;103(16):6463-6472. doi: 10.1007/s00253-019-09978-7.

23. Härtel C, Pagel J, Rupp J, et al. Prophylactic use of Lactobacillus acidophilus/Bifidobacterium infantis probiotics and outcome in very low birth weight infants. J Pediatr. 2014;165:285-289.e1. doi.10.1016/j.jpeds.2014.04.029

24. Duffield SD, Clarke P. Current use of probiotics to prevent necrotising enterocolitis. Arch Dis Child Fetal Neonatal Ed. 2019;104:F228. doi.10.1136/archdischild-2018-316199

Confidential: For Review Only

Footnotes

Contributors:

BMK: is Clinical Research Associate providing clinical, research and organizational support in the set-up and implementation of the trial; drafted and revised the manuscript

MK: contributes to the implementation and day-to-day management of the trial

DS: design and coordination of the trial

BF: is the Principal Investigator, and contributed to the design and coordination of the trial

ASR: provides research and organisational support in the microbiome theme; critically revised the protocol and manuscript.

BCU: Generated the randomization sequence, provided guidance on sample size determination as well as provision of guidance on data management and statistical analysis plan

MLJ: Edited the manuscript; reviewed the manuscript for technical and intellectual consistency

EF: oversees the Nutrition/Anthropometry workstream of the Stunting Hub; contributed to the design of the trial, and critically revised the protocol.

PH and CH: contributed to the design of the trial, critically revised the protocol and supervised drafting of the manuscript.

JPW: oversees the parasitology component of the study; contributed to the design of the trial, critically revised the protocol and supervised drafting of the manuscript.

AWW: oversees the microbiome component of the study; contributed to the design of the trial, critically revised the protocol and manuscript.

SA: is the Chief Investigator, designed the study, wrote the protocol, supervises the implementation of trial and preparation of the manuscript.

Funding: This work is supported by LSHTM through funding from the UKRI GCRF Action Against Stunting Hub. AWW and PH receive core funding support from the Scottish Government's Rural and Environment Science and Analytical Services (RESAS) division. The Sponsor for the study is LSTM, Pembroke Pl, Liverpool L3 5QA, United Kingdom.

Competing interests: The authors declare that they have no competing interests.

Consent for publication: Not required.

Ethical considerations: This study will be conducted in according to the Helsinki Declaration. The study has been approved by the National Ethics Committee for Scientific Research in Senegal and Research Ethics Committees of Liverpool School of Tropical Medicine and London School of Hygiene and Tropical Medicine. Important changes to the protocol will be communicated to the review boards and published in the final study.

Data availability statement: No data are available. Data sharing is not applicable to this manuscript as it describes a protocol. Trial is ongoing at the time of submission.

Abbreviations

AGP	Alpha-1 Acid Glycoprotein
BW	Birthweight
E. coli	*Escherichia coli*

EED	Environmental Enteric Dysfunction
LSTM	Liverpool School of Tropical Medicine
LSHTM	London School of Hygiene and Tropical Medicine

Supplementary files

1. Participant information sheet

Confidential: For Review Only

47076915_File000005_1154055812.docx

Addition to the GCRF Action Against Stunting Hub study; the Senegal Synbiotic Supplement (SENGSYN) study

Participant information sheet (PIS) for mothers/carers of newborns
English versions

WORKSTREAM: Gut Health; Senegal Synbiotic Supplement (SENGSYN) study
VERSION & DATE: Version 2.0 (12th June 2020)
AUTHOR: Stephen Allen; Babacar Faye; Doudou Sow
APPROVED BY: 1) CNERS, Senegal; 2) LSTM REC, UK
APPROVAL DATE: 1) November 11th 2020; 2) June 19th 2020
WEB LOCATION FOR ETHICS: 1) https://www.cners.sn/ 2) https://www.lstmed.ac.uk/research/research-integrity/research-ethics-committee

Workstream lead: Prof Stephen Allen; Room M-215, Department of Clinical Sciences, Liverpool School of Tropical Medicine, Pembroke Place, Liverpool L3 5QA, UK, Tel +44 (0)151 705 3752. Email: stephen.allen@lstmed.ac.uk

Principal Investigators:
Prof Faye Babacar (Lead Senegal); Dr Doudou Sow (Gut Health Lead Senegal)

Confidential: For Review Only

Revision chronology:		Signature Chief Investigator	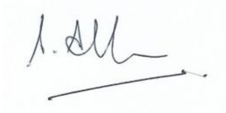
Participant Information Sheet (PIS) and consent statements (CS)	Version	Details of Changes	
PIS/CS: Parents/carers	v1.0-13th April 2020	Original	
PIS: Parents/carers	V2.0-12th June 2020	Responses to LSTM REC review (20-012), May 7th 2020 and the CNERS, Senegal REC review (SEN19/78) 23rd April 2020	
CS: Parents/carers	V2.0-12th June 2020	Separate document	

47076915_File000005_1154055812.docx

TABLE OF CONTENTS

1. Source documents in English used for subsequent translation 4
1.1. English 4
1.1.1. Participant Information Sheet for parents/carers (English) 4
1.1.2. Consent statement for screening and participation in the trial (English) 9

Confidential: For Review Only

47076915_File000005_1154055812.docx

1. SOURCE DOCUMENTS IN ENGLISH USED FOR SUBSEQUENT TRANSLATION

1.1. ENGLISH

1.1.1. Participant Information Sheet for mothers/carers (English)

Addition to the GCRF Action Against Stunting Hub study; the* Senegal Synbiotic Supplement (SENGSYN) *study

Participant Information Sheet: Mothers/carers of newborns

**Investigators**	
Prof Stephen Allen (PI)[1]	Prof Faye Babacar (Lead Senegal)[2]
Clinical Research Associate[1]: to be appointed	Assoc Prof. Doudou Sow (Gut Health lead; Senegal)[3]
**Investigators' institutions**	
1 Liverpool School of Tropical Medicine,	
2 Université Cheikh Anta DIOP, Dakar, Senegal	
3 Service de Parasitologie-Mycologie<br>Section Biologie et Explorations Fonctionnelles<br>UFR Sciences de la Santé, Senegal	

Confidential: For Review Only

47076915_File000005_1154055812.docx

Addition to the GCRF Action Against Stunting Hub study; the Senegal Synbiotic Supplement (SENGSYN) *study*

Participant Information Sheet: Mothers/carers of newborns

Introduction
As you will know, you are already part of a study to find out why some otherwise healthy children do not grow as well as expected and become "stunted". One of the factors that contributes to poor growth is an unhealthy gut. An unhealthy gut means that the baby does not breakdown and absorb food (nutrients) as well as they should and is also more prone to infections. We call this gut problem "environmental enteric dysfunction". It is caused by the baby swallowing unhealthy bacteria (harmful microbes or germs) which damage the gut.

A very important way to protect the gut in young babies is exclusive breast feeding. This is where the baby only has breast milk and no other liquids or foods. We encourage all mothers to exclusively breast feed their babies. However, despite exclusive breast feeding and the best efforts of the mother and other carers to look after their baby, we know that many babies still get an unhealthy gut.

Beneficial bacteria live in a healthy gut and help fight-off harmful microbes. Babies acquire these beneficial bacteria naturally during a normal delivery and also during the first few months of life through breastfeeding. However, there are many factors that prevent young babies acquiring these beneficial bacteria in the gut.

In this study, we want to see if we can support the growth of beneficial bacteria in the gut in young babies. This is done in many countries and leads to health benefits. In a recent large study in India, boosting the beneficial bacteria in the gut of newborn babies prevented infections. This study will assess whether this approach benefits babies in Senegal.

We invite you and your baby to take part in this study. Before you decide whether to take part, it is important for you to understand why the study is being done and what it will involve. Please take the time to read the following information carefully. If you prefer, this information can be read to you. Please do ask us if there is anything that is not clear and if you would like more information. You can decide whether or not to take part up until your baby is four days old.

What is the purpose of the study?
In this study, we want to see whether we can boost the beneficial gut bacteria in babies to improve their growth. We will also test whether boosting the beneficial bacteria prevents infections and also improves gut health.

Why has my baby been chosen?
You have already been part of the Action Against Stunting study and have delivered a healthy baby. We will invite mothers/carers with a healthy baby to partake in this study. If you agree, we will check that your baby is OK to join the study.

What intervention is being tested?

Confidential: For Review Only

47076915_File000005_1154055812.docx

The main types of beneficial bacteria that occur naturally in the gut in healthy, breastfed babies are Bifidobacteria and Lactobacilli. Many people take these bacteria as dietary supplements called “probiotics”. Another approach is to give special sugars (“prebiotics”) that support the growth of beneficial bacteria. The benefit may be greatest when a probiotic and a prebiotic are combined - called a “synbiotic”. Prebiotics, probiotics and synbiotics have been tested in many research studies in many different countries, including in very small babies and sick people, and found to be safe.

In this study, we will give babies a dietary supplement of a synbiotic. This contains 3 different strains of Bifidobacteria and Lactobacilli that are present in products that are specially designed for babies and are already used in several countries. The label provided with the synbiotic contains more information. Please do ask if you would like to see this. These details are also provided at the end of this form.

The supplement is given as a small amount of powder that can be sprinkled directly into the baby’s mouth before feeding or mixed in a clean container with expressed breast milk or sterile water.

After your baby has taken a feed well, the supplement will be given once a day for 10 days and then once weekly until your baby is 6 months old. Our research staff will either visit you at home or see you in a research clinic to help you to give the supplement.

We will compare the babies who receive the supplement with babies who do not get anything extra so we can see whether it improves growth. All babies in the study will receive special attention from the research team. This includes regular monitoring of growth and also support when babies are unwell.

What will happen if I want my baby to take part?
If you agree for your baby to join the study, your baby has a 1 in 3 chance of receiving the supplement. In other words, for every 3 babies that join the study, 1 will get the supplement and 2 will not receive anything extra. It is important that babies in the study do not take probiotic or synbiotic supplements other than those provided by the study team.

You have already agreed to provide information about feeding and how you keep the baby clean, for your baby to be measured regularly and provide samples of stool and blood. Apart from the extra visits, there will be no change; no extra samples or measurements are required for this study. The main activities of the study are summarised in the following diagram. A urine sample will also be collected at 6, 18 and 24 months as part of the main study.

Confidential: For Review Only

47076915_File000005_1154055812.docx

Synbiotics in infants in Senegal; Overview

Age of baby:	Birth	1m	2m	3m	4m	5m	6m	12m	18m	24m
**Interventions:**	Arm 1	Synbiotic daily for 10 days then weekly								
	Arm 2	No supplement								
**Collection of information and samples**										
Weekly diary		√	√	√	√	√	√	√	√	√
Weight; Feeding	√	√	√	√	√	√	√	√	√	√
Measurements of length and head size		√		√			√	√	√	√
Stool sample		√					√			√
Blood sample							√			√

What will we do today?
If you agree for your baby to join the study, we will allocate the baby by chance to either receive the supplement or not. If your baby is allocated to the supplement, the staff will provide you with the first supplement and help you to give it to your baby. We will only start babies on a supplement once they have taken a feed well.

We will call you or send you telephone messages to remind you of the follow-up visits.

What else will be expected of me if I agree for my baby to be in the study?
If your baby is unwell at anytime during the study or if you think the supplement is causing any harmful effects, we would like you to contact us to let us know. You can do this by calling or texting one of the team or telling the staff at your health centre.

All information collected from you during the study will be used by staff and scientists involved in the study. This includes staff in Liverpool, UK. The information about you and your baby and the samples collected will be handled as explained in the information sheet for the main study. We will make sure that your information is kept private by removing your name and address. Your personal details will be destroyed within 10 years of the end of the study.

What are the possible disadvantages and risks of taking part?
The bacteria in the supplement in this study have been given to many babies without causing any harm. However, if you think the synbiotic causes any harmful effects in your baby, we will discuss with you if you need to stop giving it. If this happens, we would still like your baby to continue with the

Confidential: For Review Only

follow-up. All babies will be given a thorough health check and any problems will be recorded. We will stop giving the supplement if we suspect that it is causing health problems.

The extra visits by our research staff and giving the supplement will be time consuming and may interfere with your normal daily schedule. However, we will keep the amount of time required to a minimum. Should the research staff identify any health conditions in your child during the study, they will notify you of this and help with referral for appropriate assessment.

Costs and compensation for being in the study

It is free to take part in the study. Although we do not expect that taking part in this study will incur any costs for you, you will receive reimbursement if you do incur costs that are required for your baby to take part in the study. These include travel costs (likely between £3-5) and inconvenience costs if required to attend a research clinic.

What if I don't want my baby to be in the study?

You are free to choose whether or not you want your baby to be part of this study and you have the right to refuse. If you decide that your baby should not join the study, he/she will continue in the Action Against Stunting Hub study and also receive all the standard health care that babies usually receive.

If you choose for your baby to participate in the study, you are free to change your mind and withdraw him/her from the study at any time. You do not have to give a reason. Even if you withdraw from the supplement study, your baby will still get the standard care that all babies receive.

Who is funding and sponsoring this study?

The study is funded by the United Kingdom Research and Innovation Global Challenges Research Fund. The sponsor responsible for the study is the Liverpool School of Tropical Medicine, Liverpool, UK

Contact information for questions or concerns

If you have any questions about this study, wish to make a complaint or if you want your child to stop being part of the study, please contact Porf Babacar Faye, Service de Parasitologie – Mycologie, Faculté de Médecine, Pharmacie et Odontologie, Université Cheikh Anta DIOP, Dakar BP : 5005 Dakar Fann / 16 949 Dakar Fann Tel: (221) 33 825 19 98; Email: bfaye67@yahoo.fr

You can also contact any of our study staff or staff at your health centre.

If you have any questions about you or your baby's rights as study participants, or if you think you or your child has been injured because of this study, please contact <<details of the Senegal REC>>
We hope the results of this study will help to improve growth and gut health in babies in this area.

Thank you very much for your time.

You will be given a copy of this information sheet and the signed consent form to keep for your records. One copy will be kept by the study staff.

Confidential: For Review Only

47076915_File000005_1154055812.docx

Information about the synbiotic supplement: Labinic[*] Synbiotic.

This product has been manufactured for a clinical trial and should only be used according to the instructions in the study. It is completely free of dairy, lactose, gluten, colourings, stabilisers, preservatives, animal products and other allergens.

INSTRUCTIONS FOR USE

Before using this product, read and keep this leaflet. If you notice any side-effects, then please tell your health professional straight away. This is an edible, dietary supplement. It is not a medicine. It is manufactured to high standards. Each batch is monitored for contaminants. This product is designed for use in SENGSYN study and should be used according to the study instructions.

Contents

Labinic Synbiotic is presented in strips of blister-packed white/white capsules in a poly-foil bag with silica sachets. Each capsule contains a powder containing:
(Active ingredients) 5 billion (total) of *Lactobacillus acidophilus* NCFM, *Bifidobacterium bifidum* Bb-06 and *Bifidobacterium infantis* Bi-26 probiotics and BENEO Orafti® Synergy1[**]; 50 oligofructose:50 FOS; 200mg; (Inactive ingredients Ph102 microcrystalline cellulose 280mg, Magnesium stearate 10mg, Aerosil 200 Pharma 5mg).

How to take/administer Labinic Synbiotic

Remove capsule from blister pack just before use. Open the capsule and mix contents with a small amount of breastmilk in a clean container (or sterile water if baby is not breast fed) or sprinkle directly into the baby's open mouth before a feed.

Labinic Synbiotic - Possible side-effects

Dietary supplements containing live enteric bacteria are usually well tolerated. Labinic Synbiotic may occasionally cause abdominal discomfort, diarrhoea and/or flatulence. Not recommended for use where gut or general immunity is compromised. Systemic infection is possible but very rare. Do not use Labinic Synbiotic if recipient is allergic or hypersensitive to any of the ingredients. Seek urgent treatment from a doctor or a member of the research team if taking Labinic Synbiotic makes recipient feel ill or there are any concerns about rashes or infection. Responsibility for safety and monitoring rests with the research team.

Do not exceed the recommended dose. There are no reports of adverse effects from the use or overdose of these probiotics at the time of production of this information leaflet.

Storage

We recommend keeping the blister-pack in the poly-foil bag which should be stored in a cool place away from direct heat or sunlight.
Do not use after expiry date (see packaging).

KEEP OUT OF THE REACH AND SIGHT OF CHILDREN

www.biofloratech.com

[*]Labinic is a registered trademark of Biofloratech Ltd, Image Court 139, 328 - 334 Molesey Rd, Walton-on-Thames, Surrey KT12 3LT, United Kingdom
[**]Beneo Orafti® Synergy 1 is a registered trademark of BENEO GmbH, Maximilianstrasse 10, 68165 Mannheim, Germany

Confidential: For Review Only

BMJ Paediatrics Open

Improving gut health and growth in early life: a protocol for an individually randomised, two-arm, open-label, controlled trial of a synbiotic in infants in Kaffrine District, Senegal

Journal:	*BMJ Paediatrics Open*
Manuscript ID	bmjpo-2022-001629.R1
Article Type:	Protocol
Date Submitted by the Author:	20-Sep-2022
Complete List of Authors:	Momo Kadia, Benjamin; Liverpool School of Tropical Medicine Department of Clinical Sciences, Department of Clinical Sciences<br>Khouma, Marietou; Cheikh Anta Diop University of Dakar<br>Sow, Doudou; Université Gaston Berger<br>Faye, Babacar; Cheikh Anta Diop University of Dakar<br>Ramsteijn, Anouschka; University of Aberdeen, Rowett Institute of Nutrition and Health<br>Calvo-Urbano, Beatriz; University of London, Department of Pathobiology and Population Sciences<br>Jobarteh, Modou; London School of Hygiene & Tropical Medicine<br>Ferguson, Elaine; London School of Hygiene & Tropical Medicine<br>Haggarty, Paul; University of Aberdeen<br>Webster, Joanne P.; University of London, Department of Pathobiology and Population Sciences<br>Walker, Alan; University of Aberdeen,<br>Heffernan, Claire; London School of Hygiene & Tropical Medicine; London International Development Centre<br>Allen, Stephen; Liverpool School of Tropical Medicine, Department of Clinical Sciences
Keywords:	Growth, Gastroenterology, Microbiology, Parasitology, Molecular Biology

SCHOLARONE™
Manuscripts

I, the Submitting Author has the right to grant and does grant on behalf of all authors of the Work (as defined in the below author licence), an exclusive licence and/or a non-exclusive licence for contributions from authors who are: i) UK Crown employees; ii) where BMJ has agreed a CC-BY licence shall apply, and/or iii) in accordance with the terms applicable for US Federal Government officers or employees acting as part of their official duties; on a worldwide, perpetual, irrevocable, royalty-free basis to BMJ Publishing Group Ltd ("BMJ") its licensees and where the relevant Journal is co-owned by BMJ to the co-owners of the Journal, to publish the Work in this journal and any other BMJ products and to exploit all rights, as set out in our licence.

The Submitting Author accepts and understands that any supply made under these terms is made by BMJ to the Submitting Author unless you are acting as an employee on behalf of your employer or a postgraduate student of an affiliated institution which is paying any applicable article publishing charge ("APC") for Open Access articles. Where the Submitting Author wishes to make the Work available on an Open Access basis (and intends to pay the relevant APC), the terms of reuse of such Open Access shall be governed by a Creative Commons licence – details of these licences and which Creative Commons licence will apply to this Work are set out in our licence referred to above.

Other than as permitted in any relevant BMJ Author's Self Archiving Policies, I confirm this Work has not been accepted for publication elsewhere, is not being considered for publication elsewhere and does not duplicate material already published. I confirm all authors consent to publication of this Work and authorise the granting of this licence.

Confidential: For Review Only

Improving gut health and growth in early life: a protocol for an individually randomised, two-arm, open-label, controlled trial of a synbiotic in infants in Kaffrine District, Senegal

Authors:

Benjamin Momo Kadia[1], Marietou Khouma[2], Doudou Sow[3], Babacar Faye[2], Anouschka S. Ramsteijn[4], Beatriz Calvo-Urbano[5], Modou L. Jobarteh[6], Elaine Ferguson[6], Paul Haggarty[4], Joanne P. Webster[5], Alan W. Walker[4], Claire Heffernan[5,6,7], Stephen J Allen[1]

Authors' affiliations

1. Department of Clinical Sciences, Liverpool School of Tropical Medicine (LSTM), Liverpool, United Kingdom (UK)
2. Service de Parasitologie-Mycologie, Faculté de Médecine, Université Cheikh Anta Diop (UCAD), Dakar, Senegal
3. Service de Parasitologie-Mycologie, UFR Sciences de la Santé, Université Gaston Berger, Saint Louis, Sénégal
4. Rowett Institute, University of Aberdeen, Aberdeen, UK
5. Department of Pathobiology and Population Sciences, Royal Veterinary College, University of London, Herts, AL9 7TA, UK
6. London School of Hygiene and Tropical Medicine (LSHTM), London, UK
7. London International Development Centre (LIDC), London, UK

Authors' emails

BMK: benjamin.kadia@lstmed.ac.uk
MK: khoumarita93@gmail.com
DS: doudou.sow@ugb.edu.sn
BF: babacar2.faye@ucad.edu.sn
ASR: anouschka.ramsteijn@abdn.ac.uk
BCU: bcalvo@rvc.ac.uk
MLJ: modou.jobarteh@lshtm.ac.uk
EF: elaine.ferguson@lshtm.ac.uk
PH: p.haggarty@abdn.ac.uk
JPW: jowebster@rvc.ac.uk
AWW: alan.walker@abdn.ac.uk
CH: Claire.Heffernan@lidc.ac.uk
SA: stephen.allen@lstmed.ac.uk

Corresponding author

Benjamin Momo Kadia; LSTM; email: benjamin.kadia@lstmed.ac.uk

https://mc.manuscriptcentral.com/bmjpo

Confidential: For Review Only

Abstract

Introduction: Infants exposed to enteropathogens through poor sanitation and hygiene can develop a subclinical disorder of the gut called environmental enteric dysfunction (EED), characterised by abnormal intestinal histology and permeability. EED can contribute to stunting through reduced digestion and absorption of nutrients, increased susceptibility to infections, increased systemic inflammation and inhibition of growth hormones. EED can be apparent by age 12 weeks, highlighting the need for early intervention. Modulating the early life gut microbiota using synbiotics may improve resistance against colonisation of the gut by enteropathogens, reduce EED and improve linear growth.

Methods and analysis: An individually randomised, two-arm, open-label, controlled trial will be conducted in Kaffrine District, Senegal. Infants will be recruited at birth and randomised to either receive a synbiotic containing two *Bifidobacterium* strains and one *Lactobacillus* strain, or no intervention, during the first 6 months of life. The impact of the intervention will be evaluated primarily by comparing length-for-age z score at 12 months of age in infants in the intervention and control arms of the trial. Secondary outcome variables include biomarkers of intestinal inflammation, intestinal integrity and permeability, gut microbiota profiles, presence of enteropathogens, systemic inflammation, growth hormones, epigenetic status, and episodes of illness during follow-up to age 24 months.

Discussion: This trial will contribute to the evidence base on the use of a synbiotic to improve linear growth by preventing or ameliorating EED in a low-resource setting.

Ethics and Dissemination: This trial has been approved by the National Ethics Committee for Scientific Research in Senegal and the Ethics Committees of Liverpool School of Tropical Medicine (LSTM) and London School of Hygiene and Tropical Medicine (LSHTM). Key stakeholders will be informed of the findings and the results submitted for presentation at national and international conferences and for publication in peer-reviewed journals.

Trial Registration: Pan African Clinical Trials Registry; PACTR202102689928613 (https://pactr.samrc.ac.za/TrialDisplay.aspx?TrialID=14491)

Confidential: For Review Only

Summary box

What is known about the subject?

- Environmental enteric dysfunction (EED) reduces the digestion and absorption of nutrients, and increases susceptibility to infections and systemic inflammation, contributing to growth faltering in children.
- EED can appear as early as 12 weeks of age, and measures including exclusive breastfeeding, nutritional supplements and improved water, sanitation and hygiene have not been sufficient to prevent EED and subsequent growth faltering in at-risk populations.
- Supplementation with a synbiotic may enhance colonisation resistance against enteropathogens and improve gut health.

What this study hopes to add:

- Evidence of whether administration of a synbiotic in early life improves linear growth in infants in a community exposed to poor hygiene and sanitation.
- Evidence of the effect of a synbiotic on biomarkers of gut health, enteropathogen colonisation, systemic inflammation, epigenetic status, and growth.

Confidential: For Review Only

Introduction

Globally, stunting affects about 149.2 million children under five years of age, with 40% of affected children residing in Africa [1]. Stunting is a complex process that manifests physically as significantly impaired linear growth, defined by a length-for-age/height-for-age z-score more than two standard deviations below the World Health Organization Child Growth Standards median [2,3].

Recent large cluster randomised trials in Bangladesh [4], Kenya [5] and Zimbabwe [6] reported a limited impact of water, sanitation, and hygiene (WASH) interventions and provision of food supplements on linear growth. Several studies have, however, demonstrated that growth faltering is associated with an enteropathy termed environmental enteric dysfunction (EED) [7, 8]. EED is a subclinical disorder that results from damage to the intestinal mucosa by pathogenic microbes [7,8]. It is characterised by villous atrophy, intestinal inflammation, and a 'leaky' intestinal mucosa [9]. The consequences are reduced nutrient digestion and absorption, and systemic inflammation that reduces the production of growth hormones [10,11]. EED can occur as early as age 12 weeks, and despite exclusive breastfeeding [10]. Biomarkers in stool and blood have been proposed as non-invasive and accessible indices of EED [9].

One potential target for novel interventions to prevent or reduce stunting is the diverse and numerous collections of microbes ('microbiota') that colonises the gut of infants. The gut microbiota is critical for the development of the gut and other organs, mucosal and systemic immunity, and protection against gastrointestinal infections through a process termed 'colonisation resistance' [12–14]. However, the development of the gut microbiota in early life can be perturbed by factors such as caesarean section delivery and exposure to pathogens and antibiotics [15].

Synbiotics, which are a combination of (a) prebiotic(s) and (b) probiotic(s), are one type of intervention with microbiota-modulating potential. Prebiotics are substrates that selectively promote the growth and activity of beneficial host microorganisms, thereby conferring a health benefit [16]. Probiotics are live microorganisms which, when administered in adequate amounts, can confer health benefits to the host [17]. In a large trial in India, a synbiotic given for just seven days in newborns, nearly all of whom were exclusively breastfed, significantly reduced sepsis, pneumonia, and skin infections [18]. This landmark study showed that synbiotic administration is acceptable to mothers, feasible at scale and may have important health benefits. However, the study did not report effects on gut health or linear growth.

The primary objective of this trial is to determine whether supplementation with a synbiotic during the first six months of life improves linear growth of children at age 12 months. The trial will also assess the effects of the synbiotic on biomarkers of intestinal inflammation and integrity, systemic inflammation, gut microbiota maturation, levels of growth hormones, gut colonisation with enteropathogens, host epigenetic status relevant to gut and general health and growth, episodes of illness, and linear growth up to age 24 months.

Confidential: For Review Only

Methods and analysis

Trial design

SENeGal SYNbiotic (SENGSYN) is an individually randomised, two-arm, open-label, controlled trial. The protocol for this trial has been developed in accordance with the Standard Protocol Items: Recommendations for Interventional Trials (SPIRIT) guideline. The trial is part of the research conducted within the UK Research and Innovation Global Challenges Research Fund (UKRI GCRF) AASH (Action Against Stunting Hub) (https://actionagainststunting.org/) – an interdisciplinary research consortium investigating the precursors of child stunting to inform programmes and policies to reduce the global burden of this condition. Infants born to women recruited during pregnancy in Kaffrine, Senegal in the AASH observational study will be assessed for eligibility and recruited to participate in the SENGSYN trial.

Recruited infants will be randomised to receive either a daily supplement of a synbiotic in the first ten days of enrolment and then weekly up to 6 months of age, or no intervention, with the children followed up to 24 months of age. Detailed anthropometry will be conducted at birth and months 3, 6, 12, 18 and 24, stool samples will be collected at 1, 6 and 24 months, and blood samples will be collected at 6 and 24 months. Assessments of morbidity and breastfeeding are conducted weekly up to 24 months of age (Table 1).

Confidential: For Review Only

Table 1: Table of the schedule of enrolment, intervention, and assessments. The trial follows the SPIRIT (Standard Protocol Items: Recommendations for Interventional Trials) protocol. m=months

	STUDY PERIOD										
	**Enrolment/ Allocation**	**Post-allocation**									**Close-out**
**TIMEPOINT**	Birth	1m	2m	3m	4m	5m	6m	9m	12m	18m	24m
**ENROLMENT**											
**Eligibility screen**	X										
**Informed consent**	X										
**Random allocation**	X										
**INTERVENTION**											
**Intervention arm**		Synbiotic daily for 10 days then weekly till 6 months									
**Control arm**		Routine care									
**ASSESSMENTS**											
**Weekly morbidity diary**		X	X	X	X	X	X	X	X	X	X
**Body weight, breastfeeding**	X	X	X	X	X	X	X	X	X	X	X
**Other anthropometry**		X		X			X	X	X	X	X
**Stool pathogens/ microbiota/biomarkers**		X					X				X
**Blood biomarkers**							X				X
**Epigenetic status in saliva**		X									X

Study setting

The trial is based in Kaffrine District, Senegal. The total population is 257,696 inhabitants. The population of children under 23 month is 17,780 and the prevalence of low birth weight is 17%. Data from 2015 indicated a prevalence of stunted children under five of 26.8% [19]. The district has 28 health posts or clinics and 32 health huts administered by the Kaffrine Health Centre. The AASH will recruit participants in seven clinics, and women participating in the AASH study will be targeted for the recruitment of their newborns in the SENGSYN study. More details on the study setting will be provided in the supplement paper on the overview of study design, data collection and management procedures of the AASH.

Participants

Pregnant women recruited into the AASH observational study will be given an information sheet about this synbiotic trial at recruitment (supplemental file 1). The women will be further approached by study staff at health facilities or in their homes within 3 days of delivery to determine whether their newborn meets the inclusion criteria and if she/he does, to obtain informed consent for participating in the SENGSYN trial (see supplementary file 2)

Confidential: For Review Only

Inclusion criteria are:

- Singleton newborn
- Birthweight (BW) or current weight (if BW not known) ≥2000 g
- Healthy infant who is breastfed and has taken at least one breastfeed well
- Age 1-3 days

Exclusion criteria are:

- Multiple birth (e.g., twins, triplets, etc.)
- Presence of any acute illness in the newborn (e.g., fever, receiving treatment with antibiotics)
- Congenital abnormality that might be life-threatening or impair growth
- Infant with potential contraindication to synbiotic (e.g., suspected immune suppression, cardiac abnormality)
- Mother unlikely to stay in study area for the duration of the trial
- Any health or study staff concerns regarding safety to participate in the trial

Infants of women known to be HIV positive and without known immunosuppression are eligible to participate in the trial.

Randomisation and allocation

The trial statistician will prepare a computer-generated random allocation sequence using R-coding, with blocks of random size stratified by clinic, allocating newborns to one of two study arms:

Arm 1: Labinic synbiotic daily for 10 days and then weekly to age 6 months

Arm 2: Control group; usual care with no supplement

Participants (infants) will be allocated at a ratio of 1:2 in the intervention and control arms respectively. The allocation sequence will be held by an independent pharmacist in Senegal who will prepare sequentially numbered, sealed, opaque envelopes according to the random allocation sequence. The random allocation sequence will be concealed from all other members of the research team including staff allocating newborns to the trial arms and laboratory staff analysing the biological samples. Each envelope will be opened sequentially following recruitment of each newborn and will contain a card indicating the trial arm. The trial arm will then be linked to the infant's unique AASH identification number. Mothers/carers who decline for their infant to participate in the SENGSYN study will continue in the AASH observational cohort.

Following participant allocation, study staff will assist the mother/carer in administering the first dose of the synbiotic to infants in the intervention arm.

Intervention

The trial will explore the impact of the Labinic synbiotic, composed of a prebiotic (BENEO Orafti Synergy1; 50% oligofructose/50% fructooligosaccharide; 200 mg) plus three live bacterial strains (*Lactobacillus acidophilus* NCFM, *Bifidobacterium infantis* Bi-26 and *Bifidobacterium bifidum* Bb-06; total of 5 billion organisms/dose (Biofloratech Ltd, Walton-on-Thames, Surrey, UK). The synbiotic supplement is a powder in capsules with one dose per capsule. The contents of the capsule can be sprinkled directly into the infant's open mouth before feeding. Study staff will visit daily for 9 days after the initial dose and then weekly to age 6 months (32 doses in total) to supervise the administration of the synbiotic and record adherence. Administration will be repeated once if the infant vomits within 30 minutes of synbiotic administration. In infants taking antibiotics, including

Confidential: For Review Only

HIV-exposed infants taking daily co-trimoxazole, the synbiotic will be administered where possible at least 4 hours before/after the antibiotic or between doses to minimise the effect of the antibiotic on the synbiotic microbes. Antibiotic usage will be recorded during dosing visits and morbidity follow-up. Infants randomised to the control arm will receive the same follow-up as infants in the AASH observation cohort. All infants will receive routine care as per national guidelines and will have contact details and mobile phone numbers of study staff for assistance in case of infant illness.

Outcomes

All infants will be followed up to 24 months of age with anthropometry, biological sample collections (blood, stool and saliva), and morbidity assessment conducted at scheduled time points (Table 1).

Primary outcome

The primary outcome of the trial is linear growth at age 12 months assessed by length-for-age z score. Regularly calibrated equipment for anthropometry will include a Model 876 SECA/infant scale, UNICEF stadiometer/infantometer, Lufkin WP601 measuring tape and Holtain skinfold caliper. Measurements will be conducted in duplicate with a third measurement taken if the first two measurements do not agree within a specified amount. Weight will be recorded to the nearest 100 g and height, length, mid upper arm circumference (MUAC), head circumference, triceps and sub-scapular skinfolds, and knee-to- heel length to the nearest 0.1 cm. Measurements will be used to determine z-scores using the WHO growth standards for length-for-age (LAZ), weight-for-length (WLZ), triceps-for-age and sub-scapular-for-age. Children will be classified as stunted or wasted if their LAZ or WLZ is <-2SD, respectively.

Secondary outcomes

The secondary outcomes are impact of synbiotic consumption on the presence of enteropathogens and parasites in stools, biomarkers of gut health in stool and blood, biomarkers of systemic inflammation, growth hormones, faecal microbiota composition, and epigenetics markers. Stool samples collected at 1, 6 and 24 months will be analysed using multiple methods including bacterial culture, qPCR (quantitative polymerase chain reaction) and ELISA (enzyme linked immunosorbent assay) for the following:

a) Enteropathogens: *Salmonella, Shigella,* pathogenic *Escherichia coli*
b) Enteroparasites: helminths (*Ascaris lumbricoides*, *Ancylostoma duodenale*, *Necator americanus*, *Trichuris trichiura*, *Strongyloides stercoralis)*, and protozoans (*Giardia lamblia*, *Cryptosporidia parvum/hominis* and *Entamoeba histolytica/dispar)*
c) Intestinal inflammation: myeloperoxidase
d) Intestinal permeability: α_1-antitrypsin
e) Maturation of gut microbiota, assessed using Illumina-based 16S rRNA gene amplicon sequencing

Blood samples collected at 6 and 24 months will be analysed for:

a) Chronic inflammation: alpha-1-acid glycoprotein (AGP)
b) Acute inflammation: C-reactive protein
c) Gut mucosal integrity: intestinal fatty acid binding protein
d) Growth hormones: insulin-like growth factor (IGF)-1 and its carrier protein IGF binding protein 3

https://mc.manuscriptcentral.com/bmjpo

Confidential: For Review Only

Saliva samples collected within one month of birth and at 18-24 months will be analysed for epigenetic status including markers of gut and general health and growth.

Procedures for the transportation, storage and handling of the above biological samples will be detailed in other protocols of the supplement (for blood and stool samples, see protocol titled 'Assessment of the role of gut health in childhood stunting in a multi-site, longitudinal study in India, Indonesia, and Senegal: a UKRI GCRF Action Against Stunting Hub protocol paper' and for saliva samples, see epigenetics protocols and methods).

Infants who develop adverse events that may be due to the study intervention will be identified by parents/carers contacting study staff or at follow-up visits. If appropriate, participants will be referred to the hospital for evaluation and treatment according to local guidelines. In the case of a serious adverse event (SAE), subjects will be referred to an appropriate health facility for management. All SAEs will be reported to the in-country principal investigator or an assigned representative within 24 hours of the research staff becoming aware of it. The information reported will include the nature of the event, date of onset, severity, corrective therapies given, outcome and causality (i.e. unrelated, unlikely, possible, probably, definitely). The responsible study clinician will assign the causality of the event. SAEs that are unexpected and are at least 'possibly related' to the study intervention will require expedited reporting within 24 hours of the in-country principal investigator or assigned representative becoming aware of it. This will be a maximum of 48 hours after the event occurred or the study team were made aware of the event (including the 24 hours required for the field staff to report to the principal investigator/representative). Additional information will be sent within 14 additional days (full SAE report) if the reaction had not resolved at the time of e-mail notification.

Sample size determination

The sample size calculation is based on observing a clinically meaningful improvement in linear growth in the intervention arm. In the WASH Benefits study in Kenya, the control group (n=1,431) at median age 12 months (range 2-18 months) had a mean length-for-age z score (LAZ) of -1.13 (SD=1.13)[5]. To observe a 25% improvement in LAZ at age 12 months (i.e., LAZ = -0.8475; SD=1.13) with 80% power, α =0.05 and with 1:2 group allocation, would require 189 infants in the intervention group and 378 in the control group. We plan to allow for 20% loss to follow-up by recruiting 236 to the intervention group and 472 to the control arm. Based on previous studies, we consider that this number of infants will also be sufficient to observe meaningful differences in biomarkers between the study arms. [8,10,12,20].

Data management and statistical analysis

Data and sample management and governance

Data and sample management will follow procedures established by the AASH. This includes a data sharing strategy that will build upon the guiding principles for scientific management and stewardship and Concordat on Open Research Data to ensure equitable access to data. Data will be transmitted via secure file transfer protocols between Senegal and hub partners in the UK, Indonesia, and India. A data monitoring and evaluation committee (DMEC), a trial steering committee (TSC), research ethics committees of LSTM, and LSHTM, and the national ethics committee for scientific research in Senegal will oversee the conduct of the trial. The trial sponsor, LSTM, will review the trial at initiation, during the study and at trial closeout with additional

Confidential: For Review Only

monitoring visits as required. The trial may be stopped or suspended by the sponsor at any stage due to any arising safety concerns or following t recommendation of the DMEC and/or TSC.

Statistical analysis

Analysis of the primary and secondary endpoints will be based on intention to treat. Mean/median values of biomarkers, the proportion of infants with abnormal biomarker concentrations, gut colonisation with enteropathogens and the proportion of infants/children with episodes of illnesses will be compared in the two arms at 1, 6 and 24 months. Additional analysis of the primary and secondary endpoints will also be presented according to adherence to synbiotic administration. Data will be analysed using generalised linear models with treatment as the only predictor, generating the estimates of treatment effects and their 95% confidence intervals. For outcomes with repeated measurements, the linear model will have a binomial distribution and logit link function for binary outcomes and a normal distribution and identify link function for continuous outcomes. Mean differences between the two treatment arms together with 95% confidence intervals will be derived from the appropriate linear model. Missing data will be treated as missing completely and no imputation of primary or secondary endpoints will be made. Adjustment for co-variates and inclusion of interaction terms will be performed as appropriate. More details on statistical analysis methods using secondary variables will be described in other papers in the supplement.

Patient and public involvement

There was no involvement of patients or the public in the design of this trial.

Protocol version

Version 2.0 of 12 June 2020

Discussion and conclusion

Childhood stunting affects many children in vulnerable populations, particularly in Africa, while interventions targeting WASH and nutrition have had limited success improving linear growth. Evidence has accumulated for suboptimal gut health and EED contributing to early life growth faltering. This clinical trial aims at evaluating the effectiveness of a synbiotic in improving linear growth in early life, as a potential affordable and scalable public health intervention.

There are many potential pre-, pro- and synbiotic preparations on the market, with different mixtures of substrates and microorganisms. However, the limited knowledge of their effects on the gut complicates the selection of specific products for evaluation in clinical trials, including for the prevention or amelioration of EED. Identifying probiotics with specific anti-pathogen properties relevant to EED is difficult given the limitations of *in vitro* and animal models in mimicking the complex environment of the infant's gut, and the numerous enteropathogens that may contribute to EED [10]. Colonisation resistance is mediated through multiple mechanisms, and some are likely shared amongst *Lactobacillus* and *Bifidobacterium* species, including production of fermentation acids and acidifying the gut lumen [15]. Probiotics may have additional beneficial effects in EED through reducing intestinal inflammation and permeability [21]. Prebiotics may supplement human milk oligosaccharides, the natural prebiotics in breast milk, with additional carbon sources to promote the growth of putatively beneficial microbes such as bifidobacteria [22]. Prebiotics may also serve as decoy receptors for enteropathogens [23].

Confidential: For Review Only

Lactobacilli and bifidobacteria are supplied routinely to about 70% of preterm infants nursed in neonatal units in Germany [24], based on their potential to prevent necrotising enterocolitis [18] and late onset sepsis [5]. A recent survey reported that Labinic was used routinely in preterm infants in seven neonatal units in the UK [25]. The Labinic probiotic has been associated with a significant decrease in necrotising enterocolitis and late onset sepsis in preterm infants with no episodes of sepsis due to *Lactobacillus* or *Bifidobacterium* [20]. In addition to evaluation in the SENGSYN trial, we are liaising with the research team of the on-going PROSYNK study in western Kenya (Pan Africa Clinical Trial Registry; PACTR202003893276712) to allow a comparison of the effects of the Labinic synbiotic in two different settings.

Despite the widespread and apparently safe use of probiotics in highly susceptible individuals, adverse effects of an administered *Lactobacillus* have been reported in sick children receiving intensive care [21]. Although we are not recruiting sick children in this study, we will closely monitor serious adverse events.

We will start the intervention within the first 3 days of life to limit competition from other gut organisms that rapidly start to colonise the gut following birth. Administration will discontinue at 6 months as the introduction of complementary food is associated with a marked increase in diversity of the gut microbiota [14] likely reducing the effects of pre-, pro- and synbiotics.

The same procedures that will be undertaken in all infants participating in the AASH cohorts, including the assessment of feeding and WASH practices, epigenetic status, and characterisation of the gut microbiota, will also be undertaken in infants receiving the synbiotic in this study. This will provide a valuable opportunity to explore how synbiotic administration interacts with other domains and the mechanisms through which it may affect growth.

The SENGSYN study will establish an infrastructure in rural Senegal for evaluating alternative pre-, pro- and synbiotic products and other potential approaches to modulate the gut microbiota in early life. This study will also further develop our understanding of the utility of the selected biomarkers in the evaluation of EED, which may allow us to test a series of novel interventions efficiently in advance of large-scale clinical trials.

Confidential: For Review Only

References

1. World Health Organization, World Bank, United Nations International Children's Emergency Fund. Levels and trends in child malnutrition: Key findings of the 2020 Edition of the Joint Child Malnutrition Estimates. Available at https://www.who.int/publications-detail-redirect/9789240025257

2. Prendergast AJ, Humphrey JH. The stunting syndrome in developing countries. Paediatr Int Child Health. 2014;34:250–65. doi.10.1179/2046905514Y.0000000158

3. de Onis M, Branca F. Childhood stunting: A global perspective. Matern Child Nutr. 2016;12:12–26. doi.10.1111/mcn.12231

4. Budge S, Parker AH, Hutchings PT, Garbutt C. Environmental enteric dysfunction and child stunting. Nutr Rev. 2019;77:240–253. doi.10.1093/nutrit/nuy068

5. Null C, Stewart CP, Pickering AJ, et al. Effects of water quality, sanitation, handwashing, and nutritional interventions on diarrhoea and child growth in rural Kenya: a cluster-randomised controlled trial. Lancet Glob Heal. 2018;6:e316–29. doi.org/10.1016/S2214-109X(18)30005-6

6. Luby SP, Rahman M, Arnold BF, et al. Effects of water quality, sanitation, handwashing, and nutritional interventions on diarrhoea and child growth in rural Bangladesh: a cluster randomised controlled trial. Lancet Glob Heal. 2018;6:e302–15. doi.org/10.1016/S2214-109X(17)30490-4

7. Kosek MN, Ahmed T, Bhutta ZA, et al. Causal Pathways from Enteropathogens to Environmental Enteropathy: Findings from the MAL-ED Birth Cohort Study. EBioMedicine. 2017;18:109–117. doi.10.1016/j.ebiom.2017.02.024

8. George CM, Burrowes V, Perin J, et al. Enteric Infections in Young Children are Associated with Environmental Enteropathy and Impaired Growth. Trop Med Int Heal. 2018;23:26–33. doi.10.1111/tmi.13002

9. Tickell KD, Atlas HE, Walson JL. Environmental enteric dysfunction: A review of potential mechanisms, consequences and management strategies. BMC Med. 2019;17:1–9. doi.10.1186/s12916-019-1417-3

10. Naylor C, Lu M, Haque R, et al. Environmental Enteropathy, Oral Vaccine Failure and Growth Faltering in Infants in Bangladesh. EBioMedicine. 2015;2:1759–1766. doi.10.1016/j.ebiom.2015.09.036

11. Prendergast AJ, Kelly P. Interactions between intestinal pathogens, enteropathy and malnutrition in developing countries. Curr Opin Infect Dis. 2016;29:229–36. doi.10.1097/QCO.0000000000000261

12. Arrieta MC, Stiemsma LT, Amenyogbe N, Brown E, Finlay B. The intestinal microbiome in early life: Health and disease. Front Immunol. 2014;5:47. doi.10.3389/fimmu.2014.00427

13. Buffie CG, Pamer EG. Microbiota-mediated colonization resistance against intestinal pathogens.

Confidential: For Review Only

Nat Rev Immunol. 2013;13:790–801. doi.10.1038/nri3535

14. Shanahan F, Van Sinderen D, O'Toole PW, Stanton C. Feeding the microbiota: Transducer of nutrient signals for the host. Gut. 2017;66:1709–17. doi.10.1136/gutjnl-2017-313872

15. Vandenplas Y, Carnielli VP, Ksiazyk J, et al. Factors affecting early-life intestinal microbiota development. Nutrition. 2020;78:110812. doi.10.1016/j.nut.2020.110812

16. Gibson GR, Hutkins R, Sanders ME, et al. Expert consensus document: The International Scientific Association for Probiotics and Prebiotics (ISAPP) consensus statement on the definition and scope of prebiotics. Nat Rev Gastroenterol Hepatol. 2017;14:491–502. doi.10.1038/nrgastro.2017.75

17. Food and Agriculture Organization (FAO)/World Health Organization (WHO). Guidelines for the Evaluation of Probiotics in Food. London, Ontario, Canada; 2002. Available from: http://www.who.int/foodsafety/publications/fs_management/probiotics2/en/

18. Panigrahi P, Parida S, Nanda NC, Satpathy R, Pradhan L, Chandel DiS, et al. A randomized synbiotic trial to prevent sepsis among infants in rural India. Nature. 2017;548:407–12. doi.10.1038/nature23480

19. Ibrahima Hathie. Ending Rural Hunger: The case of Senegal. Africa Growth Initiative at BROOKINGS. 2017:1–46. https://endingruralhunger.org/data/country-profiles/senegal

20. Robertson C, Savva GM, Clapuci R, Jones J, Maimouni H, Brown E, et al. Incidence of necrotising enterocolitis before and after introducing routine prophylactic Lactobacillus and Bifidobacterium probiotics. Arch Dis Child Fetal Neonatal Ed. 2020;105:380–6. doi.10.1136/archdischild-2019-317346

21. Yelin I, Flett KB, Merakou C, et al. Genomic and epidemiological evidence of bacterial transmission from probiotic capsule to blood in ICU patients. Nat Med. 2019;25:1728–32. Available from: 10.1038/s41591-019-0626-9

22. Lawson, M.A.E., O'Neill, I.J., Kujawska, M. *et al.* Breast milk-derived human milk oligosaccharides promote *Bifidobacterium* interactions within a single ecosystem. *ISME J* **14,** 635–648 (2020). https://doi.org/10.1038/s41396-019-0553-2

23. Monteagudo-Mera A, Rastall RA, Gibson GR, Charalampopoulos D, Chatzifragkou A. Adhesion mechanisms mediated by probiotics and prebiotics and their potential impact on human health. Appl Microbiol Biotechnol. 2019;103(16):6463-6472. doi: 10.1007/s00253-019-09978-7.

24. Härtel C, Pagel J, Rupp J, et al. Prophylactic use of Lactobacillus acidophilus/Bifidobacterium infantis probiotics and outcome in very low birth weight infants. J Pediatr. 2014;165:285-289.e1. doi.10.1016/j.jpeds.2014.04.029

25. Duffield SD, Clarke P. Current use of probiotics to prevent necrotising enterocolitis. Arch Dis Child Fetal Neonatal Ed. 2019;104:F228. doi.10.1136/archdischild-2018-316199

Footnotes

Contributors:

BMK: is Clinical Research Associate providing clinical, research and organizational support in the set-up and implementation of the trial; drafted and revised the manuscript

MK: contributes to the implementation and day-to-day management of the trial

DS: design and coordination of the trial

BF: is the Principal Investigator, and contributed to the design and coordination of the trial

ASR: provides research and organisational support in the microbiome theme; critically revised the protocol and manuscript.

BCU: Generated the randomization sequence, provided guidance on sample size determination as well as provision of guidance on data management and statistical analysis plan

MLJ: Edited the manuscript; reviewed the manuscript for technical and intellectual consistency

EF: oversees the Nutrition/Anthropometry workstream of the Stunting Hub; contributed to the design of the trial, and critically revised the protocol.

PH and CH: contributed to the design of the trial, critically revised the protocol and supervised drafting of the manuscript.

JPW: oversees the parasitology component of the study; contributed to the design of the trial, critically revised the protocol and supervised drafting of the manuscript.

AWW: oversees the microbiome component of the study; contributed to the design of the trial, critically revised the protocol and manuscript.

SA: is the Chief Investigator, designed the study, wrote the protocol, supervises the implementation of trial and preparation of the manuscript.

Funding: This work is supported by LSHTM through funding from the UKRI GCRF Action Against Stunting Hub (Grant reference number: MR/S01313X/1). AWW and PH receive core funding support from the Scottish Government's Rural and Environment Science and Analytical Services (RESAS) division. The Sponsor for the study is LSTM, Pembroke Pl, Liverpool L3 5QA, United Kingdom.

Competing interests: The authors declare that they have no competing interests.

Consent for publication: Not required.

Ethical considerations: This study will be conducted in according to the Helsinki Declaration. The study has been approved by the National Ethics Committee for Scientific Research in Senegal and Research Ethics Committees of Liverpool School of Tropical Medicine and London School of Hygiene and Tropical Medicine. Important changes to the protocol will be communicated to the review boards and published in the final study.

Data availability statement: No data are available. Data sharing is not applicable to this manuscript as it describes a protocol. Trial is ongoing at the time of submission.

Abbreviations

AGP	Alpha-1 Acid Glycoprotein
BW	Birthweight

E. coli	*Escherichia coli*
EED	Environmental Enteric Dysfunction
LSTM	Liverpool School of Tropical Medicine
LSHTM	London School of Hygiene and Tropical Medicine

Supplemental file

1. Participant information sheet
2. Consent form

Confidential: For Review Only

47076915_File000005_1154055812.docx

Addition to the GCRF Action Against Stunting Hub study; the Senegal Synbiotic Supplement (SENGSYN) study

Participant information sheet (PIS) for mothers/carers of newborns English versions

WORKSTREAM: Gut Health; Senegal Synbiotic Supplement (SENGSYN) study
VERSION & DATE: Version 2.0 (12th June 2020)
AUTHOR: Stephen Allen; Babacar Faye; Doudou Sow
APPROVED BY: 1) CNERS, Senegal; 2) LSTM REC, UK
APPROVAL DATE: 1) November 11th 2020; 2) June 19th 2020
WEB LOCATION FOR ETHICS: 1) https://www.cners.sn/ 2) https://www.lstmed.ac.uk/research/research-integrity/research-ethics-committee

Workstream lead: Prof Stephen Allen; Room M-215, Department of Clinical Sciences, Liverpool School of Tropical Medicine, Pembroke Place, Liverpool L3 5QA, UK, Tel +44 (0)151 705 3752. Email: stephen.allen@lstmed.ac.uk

Principal Investigators:
Prof Faye Babacar (Lead Senegal); Dr Doudou Sow (Gut Health Lead Senegal)

<table>
<tr><td colspan="2">Revision chronology:</td><td>Signature Chief Investigator</td><td>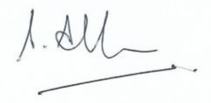</td></tr>
<tr><td>Participant Information Sheet (PIS) and consent statements (CS)</td><td>Version</td><td colspan="2">Details of Changes</td></tr>
<tr><td>PIS/CS: Parents/carers</td><td>v1.0-13th April 2020</td><td colspan="2">Original</td></tr>
<tr><td>PIS: Parents/carers</td><td>V2.0-12th June 2020</td><td colspan="2">Responses to LSTM REC review (20-012), May 7th 2020 and the CNERS, Senegal REC review (SEN19/78) 23rd April 2020</td></tr>
<tr><td>CS: Parents/carers</td><td>V2.0-12th June 2020</td><td colspan="2">Separate document</td></tr>
</table>

Confidential: For Review Only

47076915_File000005_1154055812.docx

TABLE OF CONTENTS

1. Source documents in English used for subsequent translation 4
1.1. English 4
1.1.1. Participant Information Sheet for parents/carers (English) 4
1.1.2. Consent statement for screening and participation in the trial (English) 9

Confidential: For Review Only

47076915_File000005_1154055812.docx

1. SOURCE DOCUMENTS IN ENGLISH USED FOR SUBSEQUENT TRANSLATION

1.1. ENGLISH

1.1.1. Participant Information Sheet for mothers/carers (English)

Addition to the GCRF Action Against Stunting Hub study; the* Senegal Synbiotic Supplement (SENGSYN) *study

Participant Information Sheet: Mothers/carers of newborns

**Investigators**	
Prof Stephen Allen (PI)[1]	Prof Faye Babacar (Lead Senegal)[2]
Clinical Research Associate[1]: to be appointed	Assoc Prof. Doudou Sow (Gut Health lead; Senegal)[3]
**Investigators' institutions**	
1 Liverpool School of Tropical Medicine,	
2 Université Cheikh Anta DIOP, Dakar, Senegal	
3 Service de Parasitologie-Mycologie<br>Section Biologie et Explorations Fonctionnelles<br>UFR Sciences de la Santé, Senegal	

Confidential: For Review Only

Addition to the GCRF Action Against Stunting Hub study; the Senegal Synbiotic Supplement (SENGSYN) *study*

Participant Information Sheet: Mothers/carers of newborns

Introduction

As you will know, you are already part of a study to find out why some otherwise healthy children do not grow as well as expected and become "stunted". One of the factors that contributes to poor growth is an unhealthy gut. An unhealthy gut means that the baby does not breakdown and absorb food (nutrients) as well as they should and is also more prone to infections. We call this gut problem "environmental enteric dysfunction". It is caused by the baby swallowing unhealthy bacteria (harmful microbes or germs) which damage the gut.

A very important way to protect the gut in young babies is exclusive breast feeding. This is where the baby only has breast milk and no other liquids or foods. We encourage all mothers to exclusively breast feed their babies. However, despite exclusive breast feeding and the best efforts of the mother and other carers to look after their baby, we know that many babies still get an unhealthy gut.

Beneficial bacteria live in a healthy gut and help fight-off harmful microbes. Babies acquire these beneficial bacteria naturally during a normal delivery and also during the first few months of life through breastfeeding. However, there are many factors that prevent young babies acquiring these beneficial bacteria in the gut.

In this study, we want to see if we can support the growth of beneficial bacteria in the gut in young babies. This is done in many countries and leads to health benefits. In a recent large study in India, boosting the beneficial bacteria in the gut of newborn babies prevented infections. This study will assess whether this approach benefits babies in Senegal.

We invite you and your baby to take part in this study. Before you decide whether to take part, it is important for you to understand why the study is being done and what it will involve. Please take the time to read the following information carefully. If you prefer, this information can be read to you. Please do ask us if there is anything that is not clear and if you would like more information. You can decide whether or not to take part up until your baby is four days old.

What is the purpose of the study?

In this study, we want to see whether we can boost the beneficial gut bacteria in babies to improve their growth. We will also test whether boosting the beneficial bacteria prevents infections and also improves gut health.

Why has my baby been chosen?

You have already been part of the Action Against Stunting study and have delivered a healthy baby. We will invite mothers/carers with a healthy baby to partake in this study. If you agree, we will check that your baby is OK to join the study.

What intervention is being tested?

Confidential: For Review Only

47076915_File000005_1154055812.docx

The main types of beneficial bacteria that occur naturally in the gut in healthy, breastfed babies are Bifidobacteria and Lactobacilli. Many people take these bacteria as dietary supplements called “probiotics”. Another approach is to give special sugars (“prebiotics”) that support the growth of beneficial bacteria. The benefit may be greatest when a probiotic and a prebiotic are combined - called a “synbiotic”. Prebiotics, probiotics and synbiotics have been tested in many research studies in many different countries, including in very small babies and sick people, and found to be safe.

In this study, we will give babies a dietary supplement of a synbiotic. This contains 3 different strains of Bifidobacteria and Lactobacilli that are present in products that are specially designed for babies and are already used in several countries. The label provided with the synbiotic contains more information. Please do ask if you would like to see this. These details are also provided at the end of this form.

The supplement is given as a small amount of powder that can be sprinkled directly into the baby’s mouth before feeding or mixed in a clean container with expressed breast milk or sterile water.

After your baby has taken a feed well, the supplement will be given once a day for 10 days and then once weekly until your baby is 6 months old. Our research staff will either visit you at home or see you in a research clinic to help you to give the supplement.

We will compare the babies who receive the supplement with babies who do not get anything extra so we can see whether it improves growth. All babies in the study will receive special attention from the research team. This includes regular monitoring of growth and also support when babies are unwell.

What will happen if I want my baby to take part?

If you agree for your baby to join the study, your baby has a 1 in 3 chance of receiving the supplement. In other words, for every 3 babies that join the study, 1 will get the supplement and 2 will not receive anything extra. It is important that babies in the study do not take probiotic or synbiotic supplements other than those provided by the study team.

You have already agreed to provide information about feeding and how you keep the baby clean, for your baby to be measured regularly and provide samples of stool and blood. Apart from the extra visits, there will be no change; no extra samples or measurements are required for this study. The main activities of the study are summarised in the following diagram. A urine sample will also be collected at 6, 18 and 24 months as part of the main study.

Confidential: For Review Only

47076915_File000005_1154055812.docx

Synbiotics in infants in Senegal; Overview

Age of baby:	Birth	1m	2m	3m	4m	5m	6m	12m	18m	24m
Interventions:	Arm 1	Synbiotic daily for 10 days then weekly								
	Arm 2	No supplement								
**Collection of information and samples**										
Weekly diary		√	√	√	√	√	√	√	√	√
Weight; Feeding	√	√	√	√	√	√	√	√	√	√
Measurements of length and head size		√		√			√	√	√	√
Stool sample		√					√			√
Blood sample							√			√

What will we do today?
If you agree for your baby to join the study, we will allocate the baby by chance to either receive the supplement or not. If your baby is allocated to the supplement, the staff will provide you with the first supplement and help you to give it to your baby. We will only start babies on a supplement once they have taken a feed well.

We will call you or send you telephone messages to remind you of the follow-up visits.

What else will be expected of me if I agree for my baby to be in the study?
If your baby is unwell at anytime during the study or if you think the supplement is causing any harmful effects, we would like you to contact us to let us know. You can do this by calling or texting one of the team or telling the staff at your health centre.

All information collected from you during the study will be used by staff and scientists involved in the study. This includes staff in Liverpool, UK. The information about you and your baby and the samples collected will be handled as explained in the information sheet for the main study. We will make sure that your information is kept private by removing your name and address. Your personal details will be destroyed within 10 years of the end of the study.

What are the possible disadvantages and risks of taking part?
The bacteria in the supplement in this study have been given to many babies without causing any harm. However, if you think the synbiotic causes any harmful effects in your baby, we will discuss with you if you need to stop giving it. If this happens, we would still like your baby to continue with the

Confidential: For Review Only

47076915_File000005_1154055812.docx

follow-up. All babies will be given a thorough health check and any problems will be recorded. We will stop giving the supplement if we suspect that it is causing health problems.

The extra visits by our research staff and giving the supplement will be time consuming and may interfere with your normal daily schedule. However, we will keep the amount of time required to a minimum. Should the research staff identify any health conditions in your child during the study, they will notify you of this and help with referral for appropriate assessment.

Costs and compensation for being in the study
It is free to take part in the study. Although we do not expect that taking part in this study will incur any costs for you, you will receive reimbursement if you do incur costs that are required for your baby to take part in the study. These include travel costs (likely between £3-5) and inconvenience costs if required to attend a research clinic.

What if I don't want my baby to be in the study?
You are free to choose whether or not you want your baby to be part of this study and you have the right to refuse. If you decide that your baby should not join the study, he/she will continue in the Action Against Stunting Hub study and also receive all the standard health care that babies usually receive.

If you choose for your baby to participate in the study, you are free to change your mind and withdraw him/her from the study at any time. You do not have to give a reason. Even if you withdraw from the supplement study, your baby will still get the standard care that all babies receive.

Who is funding and sponsoring this study?
The study is funded by the United Kingdom Research and Innovation Global Challenges Research Fund. The sponsor responsible for the study is the Liverpool School of Tropical Medicine, Liverpool, UK

Contact information for questions or concerns
If you have any questions about this study, wish to make a complaint or if you want your child to stop being part of the study, please contact Porf Babacar Faye, Service de Parasitologie – Mycologie, Faculté de Médecine, Pharmacie et Odontologie, Université Cheikh Anta DIOP, Dakar BP : 5005 Dakar Fann / 16 949 Dakar Fann Tel: (221) 33 825 19 98; Email: bfaye67@yahoo.fr

You can also contact any of our study staff or staff at your health centre.

If you have any questions about you or your baby's rights as study participants, or if you think you or your child has been injured because of this study, please contact <<details of the Senegal REC>>
We hope the results of this study will help to improve growth and gut health in babies in this area.

Thank you very much for your time.

You will be given a copy of this information sheet and the signed consent form to keep for your records. One copy will be kept by the study staff.

Confidential: For Review Only

Information about the synbiotic supplement: Labinic[*] Synbiotic.

This product has been manufactured for a clinical trial and should only be used according to the instructions in the study. It is completely free of dairy, lactose, gluten, colourings, stabilisers, preservatives, animal products and other allergens.

INSTRUCTIONS FOR USE

Before using this product, read and keep this leaflet. If you notice any side-effects, then please tell your health professional straight away. This is an edible, dietary supplement. It is not a medicine. It is manufactured to high standards. Each batch is monitored for contaminants. This product is designed for use in SENGSYN study and should be used according to the study instructions.

Contents

Labinic Synbiotic is presented in strips of blister-packed white/white capsules in a poly-foil bag with silica sachets. Each capsule contains a powder containing:
(Active ingredients) 5 billion (total) of *Lactobacillus acidophilus* NCFM, *Bifidobacterium bifidum* Bb-06 and *Bifidobacterium infantis* Bi-26 probiotics and BENEO Orafti® Synergy1[**]; 50 oligofructose:50 FOS; 200mg; (Inactive ingredients Ph102 microcrystalline cellulose 280mg, Magnesium stearate 10mg, Aerosil 200 Pharma 5mg).

How to take/administer Labinic Synbiotic

Remove capsule from blister pack just before use. Open the capsule and mix contents with a small amount of breastmilk in a clean container (or sterile water if baby is not breast fed) or sprinkle directly into the baby's open mouth before a feed.

Labinic Synbiotic - Possible side-effects

Dietary supplements containing live enteric bacteria are usually well tolerated. Labinic Synbiotic may occasionally cause abdominal discomfort, diarrhoea and/or flatulence. Not recommended for use where gut or general immunity is compromised. Systemic infection is possible but very rare. Do not use Labinic Synbiotic if recipient is allergic or hypersensitive to any of the ingredients. Seek urgent treatment from a doctor or a member of the research team if taking Labinic Synbiotic makes recipient feel ill or there are any concerns about rashes or infection. Responsibility for safety and monitoring rests with the research team.

Do not exceed the recommended dose. There are no reports of adverse effects from the use or overdose of these probiotics at the time of production of this information leaflet.

Storage

We recommend keeping the blister-pack in the poly-foil bag which should be stored in a cool place away from direct heat or sunlight.
Do not use after expiry date (see packaging).

KEEP OUT OF THE REACH AND SIGHT OF CHILDREN

www.biofloratech.com

[*]Labinic is a registered trademark of Biofloratech Ltd, Image Court 139, 328 - 334 Molesey Rd, Walton-on-Thames, Surrey KT12 3LT, United Kingdom
[**]Beneo Orafti® Synergy 1 is a registered trademark of BENEO GmbH, Maximilianstrasse 10, 68165 Mannheim, Germany

Confidential: For Review Only

47572584_File000002_1166351604.docx

Addition to the GCRF Action Against Stunting Hub study; the Senegal Synbiotic Supplement (SENGSYN) study

Consent statement for mothers/carers of newborns
English versions

This statement refers to the Action Against Stunting Hub SENGSYN study Participant information sheet (PIS) for mothers/carers of newborns (v2.0-12th June 2020)

Confidential: For Review Only

Consent Statement for Eligibility assessment and Participation in the study	
Your signature below means that you voluntarily agree for your baby to participate in this research	
**Assessment for eligibility for inclusion**	Please initial each box:
The above study has been explained to me and I have been given the opportunity to ask questions. I agree for my baby to be assessed to see if he/she is suitable to be in the study.	
I understand that my baby's further participation in the study will depend on the results of the eligibility assessment. I understand that my agreeing for my baby to be assessed does not mean that I have to agree for my baby to be in the study.	
I understand that I can change my mind at any time without having to give a reason and without any consequences to my baby's health care.	
**Participation in the SENGSYN study**	
I understand that I am free to choose whether or not my baby will join this study. I understand that saying "No" will allow my baby to continue in the Action Against Stunting study and not effect the usual care that my baby receives.	
I understand that there will be no change to the questionnaires, measurements and sample collection that I have already agreed to for the Action Against Stunting Hub study.	
I understand that the study team may make extra visits to me at home and in research clinics. I agree for the study team to contact me by phone and to receive mobile phone messages to remind me of the study visits.	
I have read this form and decided that I agree for my baby to take part in the study including taking the synbiotic supplement if required. The study's general objectives, details of participation and possible risks and inconveniences have been explained to me to my satisfaction. I understand that I may withdraw my consent at any time. My signature also indicates that I have received a copy of this consent form.	

47572584_File000002_1166351604.docx

ID number: _._._ _ _	**Name**	**Signature**	**Today's date**
Adult providing consent for baby			
Relationship to baby	Mother / other carer: specify ………………..……………………..……………….		
Witness*			
Study staff consenting participant			
* A witness is only needed if the participant cannot read or sign the consent form. The witness must be a person independent from the study. The participant must verbally state that he/she has had the information sheet read and explained to them and they give their consent for their baby to participate in the study in the presence of a witness who will then sign.			

Confidential: For Review Only
